# An automated 3D modeling pipeline for constructing 3D models of MONOGENEAN HARDPART using machine learning techniques

**DOI:** 10.1186/s12859-019-3210-x

**Published:** 2019-12-24

**Authors:** Bee Guan Teo, Sarinder Kaur Dhillon

**Affiliations:** 1 School of Engineering, Monash University Malaysia, Kuala Lumpur, Malaysia; 2 Data Science and Bioinformatics Laboratory, Institute of Biological Sciences, Faculty of Science, University of Malaya, Kuala Lumpur, Malaysia

**Keywords:** 3D Modelling, Machine learning, Landmark detection, NoSQL database

## Abstract

**Background:**

Studying structural and functional morphology of small organisms such as monogenean, is difficult due to the lack of visualization in three dimensions. One possible way to resolve this visualization issue is to create digital 3D models which may aid researchers in studying morphology and function of the monogenean. However, the development of 3D models is a tedious procedure as one will have to repeat an entire complicated modelling process for every new target 3D shape using a comprehensive 3D modelling software. This study was designed to develop an alternative 3D modelling approach to build 3D models of monogenean anchors, which can be used to understand these morphological structures in three dimensions. This alternative 3D modelling approach is aimed to avoid repeating the tedious modelling procedure for every single target 3D model from scratch.

**Result:**

An automated 3D modeling pipeline empowered by an Artificial Neural Network (ANN) was developed. This automated 3D modelling pipeline enables automated deformation of a generic 3D model of monogenean anchor into another target 3D anchor. The 3D modelling pipeline empowered by ANN has managed to automate the generation of the 8 target 3D models (representing 8 species: *Dactylogyrus primaries, Pellucidhaptor merus, Dactylogyrus falcatus, Dactylogyrus vastator, Dactylogyrus pterocleidus, Dactylogyrus falciunguis, Chauhanellus auriculatum* and *Chauhanellus caelatus*) of monogenean anchor from the respective 2D illustrations input without repeating the tedious modelling procedure.

**Conclusions:**

Despite some constraints and limitation, the automated 3D modelling pipeline developed in this study has demonstrated a working idea of application of machine learning approach in a 3D modelling work. This study has not only developed an automated 3D modelling pipeline but also has demonstrated a cross-disciplinary research design that integrates machine learning into a specific domain of study such as 3D modelling of the biological structures.

## Background

Studying structural and functional morphology of small organisms is difficult due to the lack of visualization in three dimensions. One viable way to resolve this is by creating digital 3D models using 3D imaging techniques such as confocal microscopy [1, 2] and scanning electron microscopy (SEM) [3–6]. As highlighted by Galli et al. [1], digital 3D models of monogenean hard parts reconstructed from the confocal microscopy contain morphological details which are not detectable in the corresponding 2D illustrations. However, 3D imaging equipment used for 3D reconstruction are expensive and demand tedious specimen preparation prior to usage [7].

Hence, Teo et al. [8] demonstrated a polygonal modelling method by using 2D illustrations as the templates to construct 3D models of haptoral parts of monogeneans via an existing commercial 3D modelling software, Autodesk 3ds Max. The resulting models produced from Autodesk 3ds Max are shown to be effective in offering 3D visualization of the spatial relationship of morphological characters in a monogenean haptor. However, Autodesk 3ds Max requires technical skill to create a decent 3D shape of a target specimen due to the high complexity of the software user interface. Another approach would be to construct a new digital 3D model from an existing 3D model via a shape deformation technique [9]. This shape deformation technique obviates the need for repeating a complicated modelling process to construct a new 3D model from scratch. One limitation of this technique is that it demands a modeller to manually choose the vertices of a digital generic 3D model and align them with the landmark points of an input 2D image. If this can be automated with minimal human intervention, the entire modelling procedure may become much easier and efficient.

A landmark point detection empowered by a machine learning algorithm could be a solution to enable auto-detection of the landmark points on an input image. Subsequently, the landmark points predicted by the machine learning model can be used to direct the deformation of a digital generic 3D model by automatically mapping its vertices with their corresponding landmark points and eventually produce a target 3D shape.

Landmark point detection has been studied extensively by researchers for tracking human facial feature points which were applied to human face recognition [10–12], facial expression analysis [13], facial landmark localization [14, 15], age estimation [16], gender classification [17] and 3D face modelling [18]. These studies share a common idea that the facial feature points located at the eye, nose, mouth, and chin of a 2D facial image carry semantic meaning and these points could automatically be detected via a machine learning algorithm using annotated 2D sample images as a training dataset. The idea of the landmark detection was also applied to several other fields of study such as geometric morphometric for bioimages [19, 20], anatomical features detection for medical diagnosis [21, 22], and human body pose detection [23].

One common machine learning algorithm used in most of the landmark detection studies is Artificial Neural Network (ANN). The popularity of ANN in recent years is due to its effectiveness for image analysis tasks [24]. In some studies, ANN was used along with a clustering algorithm to partition the training sets into several subsets which share the common properties [25] or with autoencoder to rectify the outlier points [15], and such coupling approach was able to enhance the prediction accuracy in the CNN. Autoencoder was also used on its own to automatically identify human facial expressions using some geometrical features, and the features data was trained with an unsupervised method. On the other hand, Chen et al. [12] used RNN in their landmark detection study to deal with the non-linear deformations in the human facial shape. To the best of our knowledge, there is no reported study on the application of ANN for landmark point detection to enable automated construction of digital 3D models of biological specimens such as monogeneans, which is the target specimen of this study.

Monogeneans (Class: Monogenea, Phylum: Platyhelminthes) are parasitic flatworms which possess soft anatomical structures and hard sclerotized copulatory system of both males and female reproductive parts. Their anatomical structures, such as haptoral elements are of diagnostic importance in taxonomic description and identification of the monogenean species [26–31]. However, the study of these morphological structures is not easy because monogeneans are soft-bodied flatworms with fragile endodermis, unable to withstand desiccation for long investigation under a microscope. The use of the digital 3D models would help taxonomist to understand the morphology as well as the functions of these morphological structures.

The main aim of this study is to develop an automated 3D modelling pipeline that uses 2D illustrations as input to generate a variety of digital 3D models of monogenean anchor without repeating the tedious 3D modelling process from scratch. The target models of this study are limited to the haptoral anchors of monogenean from eight selected species, which are *Dactylogyrus primaries* Gusev, 1955*, Pellucidhaptor merus* Zaika, 1961*, Dactylogyrus falcatus* Wedl, 1857*, Dactylogyrus vastator* Bybelin, 1924*, Dactylogyrus pterocleidus* Gusev, 1955*, Dactylogyrus falciunguis* Achmerow, 1952*, Chauhanellus auriculatum* Lim, 1994 and *Chauhanellus caelatus* Lim, 1994*.* This automated 3D modelling pipeline will be driven by an ANN that can detect the landmark points’ location on an input 2D illustration and automatically align them with the vertices of a generic 3D model to enable deformation of its 3D shape to produce a target 3D model.

## Results

The results obtained from the development of an automated 3D modelling pipeline are presented in the following sections.

### Synthetic 2D illustrations

**Fig. 1:**
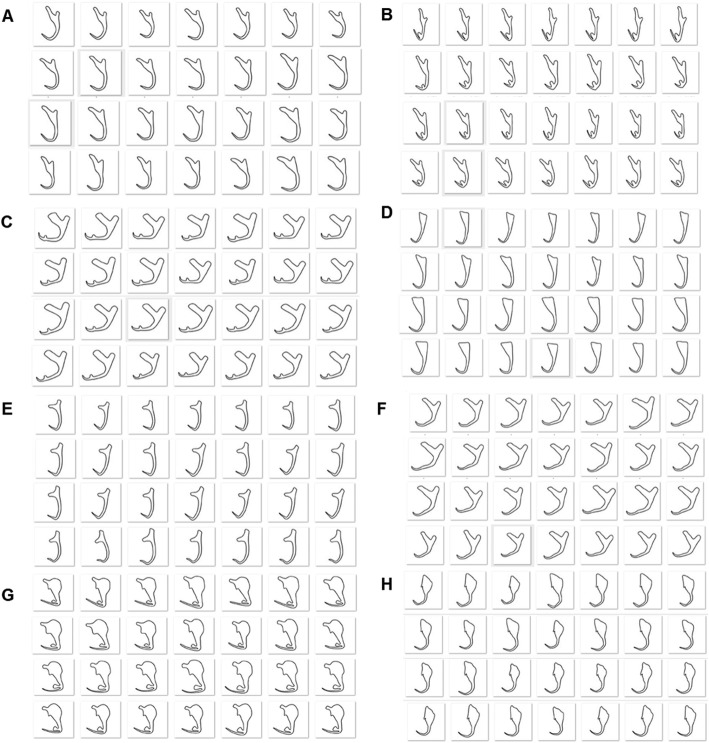
Selected samples of synthetic 2D illustrations. **a** Shape Category 1, (**b**) Shape Category 2, (**c**) Shape Category 3, (**d**) Shape Category 4, (**e**) Shape Category 5, (**f**) Shape Category 6, (**g**) Shape Category 7, (**h**) Shape Category 8

The output of data preparation includes three sets which are 1000, 2500 and 5000 synthetic 2D illustrations generated by the semi-automated augmentation program and the NoSQL database developed to store the details of the synthetic 2D illustrations. All three sets of the synthetic illustrations of monogenean anchor were synthesized based on eight different categories of morphological variant shapes to cater the need for training the machine learning model to detect the landmark points on the input illustrations of all the selected monogenean anchors. Some selected samples of the synthetic illustrations for each category of anchor shape are shown in Fig. 1a-h. The image size of all the synthetic illustrations were standardized in 96 × 96 pixels. The synthetic 2D illustrations as exemplified in Fig. 1a-h are scaled and rotated in random degrees. This is to ensure that a high degree of morphological variant shapes can be obtained to train a more generalized machine learning model.

### NoSQL database

**Fig. 2:**
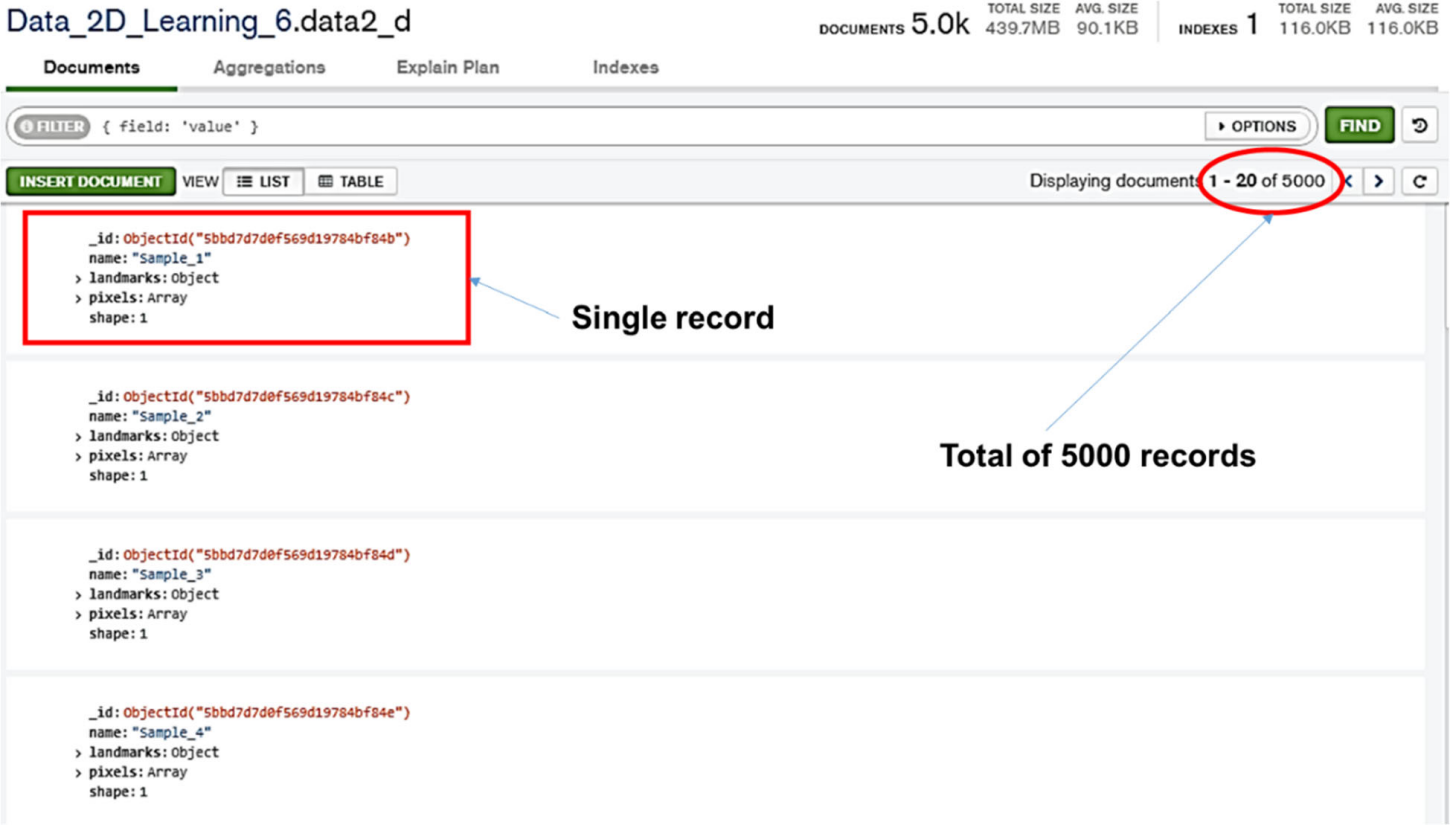
MongoDB Compass that shows details of the stored training sets in the noSQL database

**Fig. 3:**
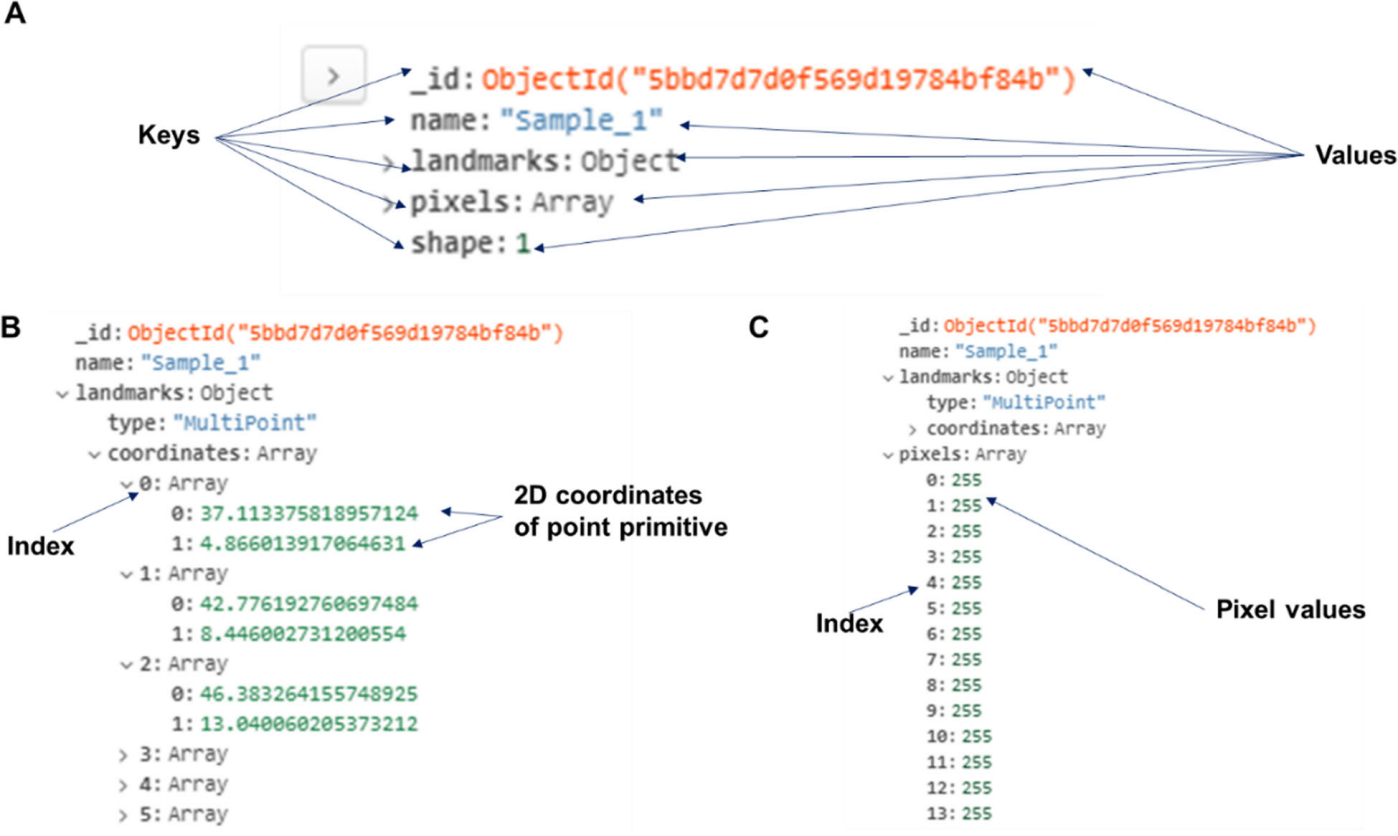
Example of data stored in noSQL database. **a** Five key-values pairs, (**b**) 2D coordinates of point primitive represented as a coordinate array, (**c**) Pixel values of synthetic illustration represented as an integer array

In this study, a NoSQL database using MongoDB was developed to store the details of all the synthetic illustration training sets such as the illustration name, pixels values of illustrations, 2D coordinates of 34 point primitives (Fig. 2). The details of the training sets are presented as five key-value pairs of data for each record (Fig. 3a). The “_id” key is auto-generated by MongoDB whenever a new record is created. The “name” and the “shape” hold the information of the illustration name and the shape category as a simple string and a numeric number respectively. The 2D coordinates of the 34 point primitives are stored in “landmark” key and they are represented as coordinate arrays (Fig. 3b). The pixel values of each illustration record is held by the “pixels” key and they are represented as an array of integer elements ranging from 0 to 255 (Fig. 3c).

### Evaluation of machine learning model based on three different datasets

The train, validation and test accuracy of the machine learning models trained by three different number of the dataset are presented in Table 1. The table shows that the model trained by 5000 datasets results in the highest test accuracy. Hence, the model trained by 5000 datasets was adopted for landmark detection on the input illustrations of eight different monogenean species.

**Table 1:** Evaluation of trained ANN model based on three different number of datasets

Number of dataset	Train Accuracy (%)	Validation Accuracy (%)	Test Accuracy (%)
1000	58.39	49.17	47.5
2500	69.93	70.33	70.4
5000	83.25	81.33	80.1

### Landmark point detection on the input illustrations of monogenean anchors using the machine learning model

**Fig. 4:**
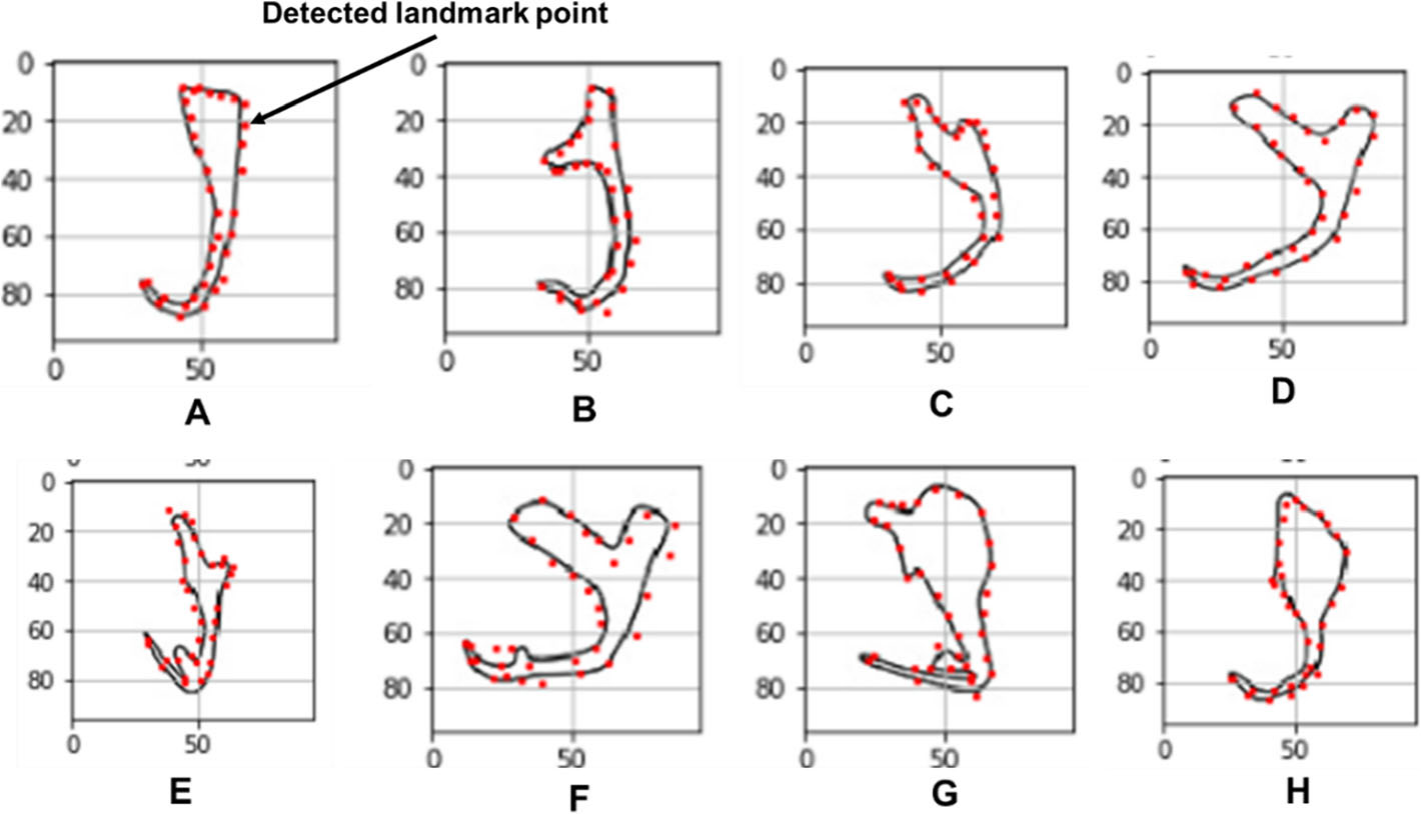
Machine detected landmarks on 2D illustrations of eight selected monogenean anchors from Gusev [43] and Lim [44]. **a** *Dactylogyrus primarius* (**b**) *Pellucidhaptor merus* (**c**) *Dactylogyrus falcatus*. **d** *Dactylogyrus vastator* (**e**) *Dactylogyrus pterocleidus* (**f**) *Dactylogyrus falciunguis* (**g**) *Chauhanellus auriculatum* (**h**) *Chauhanellus caelatus*

The machine learning model trained by the 5000 datasets was tested on the monogenean anchors of eight selected monogenean species with different shape feature to examine the versatility of the machine learning model to detect landmark point on a diverse shape of anchors (Fig. 4). In general, Fig. 4 shows favorable results of the landmark detection by having most of the landmark points (red dots) localized along the edge of the input 2D illustrations of the monogenean anchors.

### 3D models of monogenean anchors derived from deformation of the generic 3D model

**Fig. 5:**
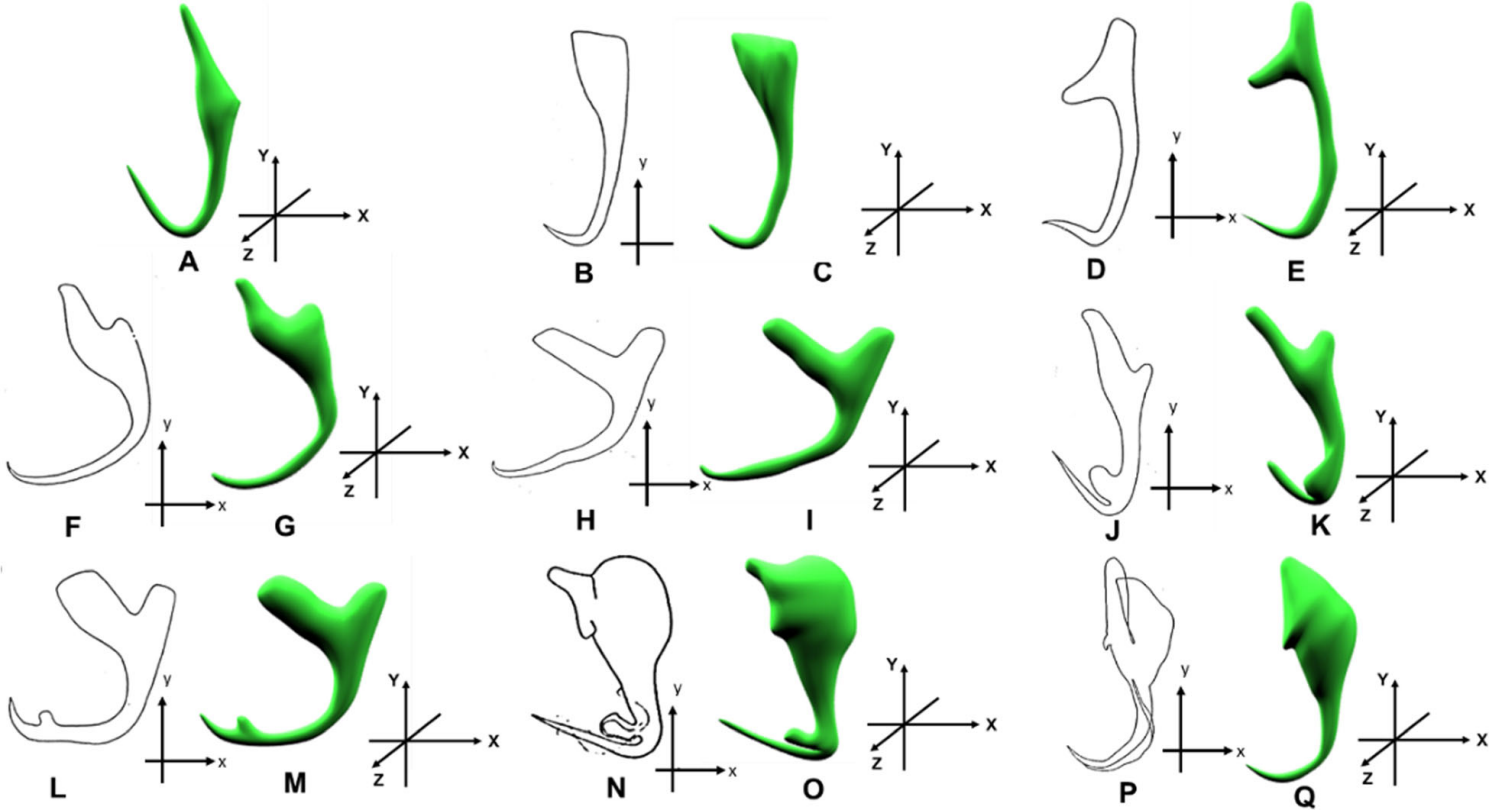
Generic 3D model and the input illustrations of anchor along with their corresponding 3D models (coloured). **a**: Generic 3D anchor model (**b**-**c**): *Dactylogyrus primarius*. **d**-**e**: *Pellucidhaptor merus*. **f**-**g**: *Dactylogyrus falcatus*. **h**-**i** *Dactylogyrus vastator*. **j**-**k**: *Dactylogyrus pterocleidus.* **l**-**m**: *Dactylogyrus falciunguis.* **n**-**o** *Chauhanellus auriculatum.* **p**-**q** *Chauhanellus caelatus*

**Fig. 6:**
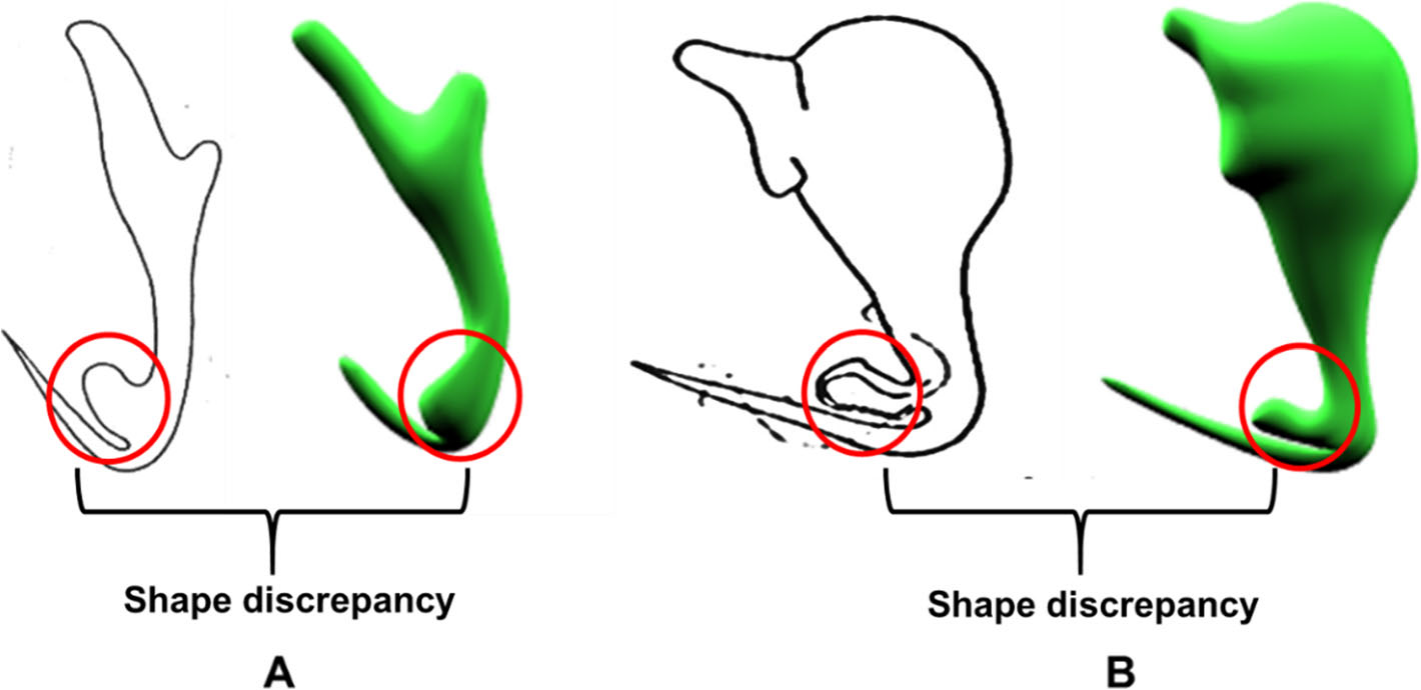
Discrepancy of 3D shape found in the extrusion part of (**a**) *Dactylogyrus peterocleidus* and (**b**) *Chauhanellus auriculatum*

In this section, the 3D models of monogenean anchors derived from the automated 3D modelling pipeline are presented. Figure 5 shows a group of 3D models (Fig. 5b-e) produced by the automated 3D modelling pipeline. In general, the overall body shape of the resulting 3D models resembles the 2D shape as presented in their corresponding 2D illustrations. Nevertheless, the automated 3D modelling pipeline does not work very well on modelling the extrusion parts of few anchors (Fig. 5k, & o). A closer inspection on the shape discrepancy presented by those anchors is shown in Fig. 6. The automated 3D modelling pipeline shows its limitation to derive the fan-like extrusion structure found in the anchors of *D. peterocleidus* and *C. auriculatum*.

### Evaluation of the efficiency of the automated 3D modelling pipeline

**Fig. 7:**
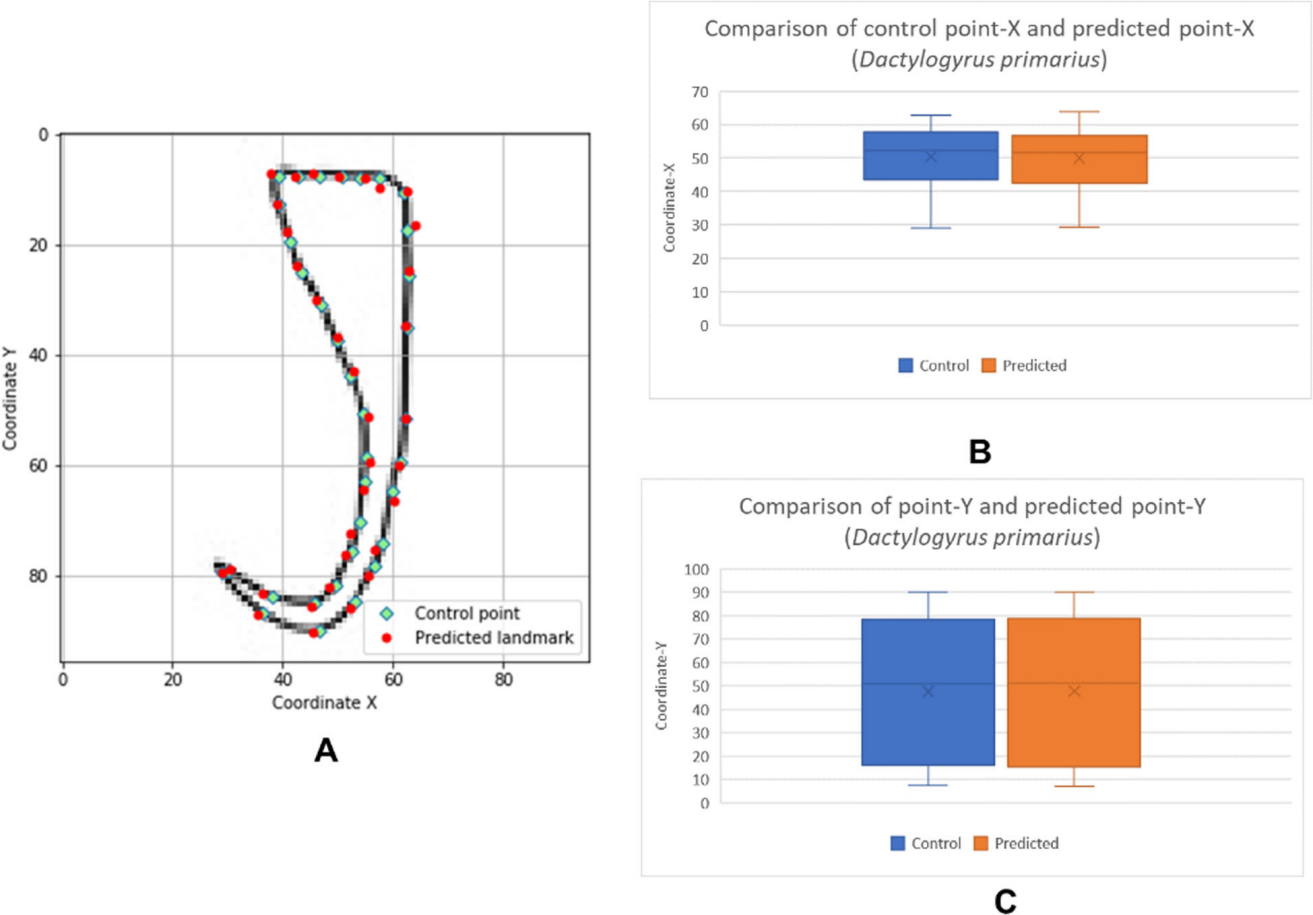
Comparison of control points and predicted points on 2D illustration of *Dactylogyrus primarius*. **a** Positions of control points (small green markers) and predicted points (red markers) on 2D illustration. **b** Boxplot for control point-X and predicted point-X (**c**) Boxplot for control point-Y and predicted point-Y

**Fig. 8:**
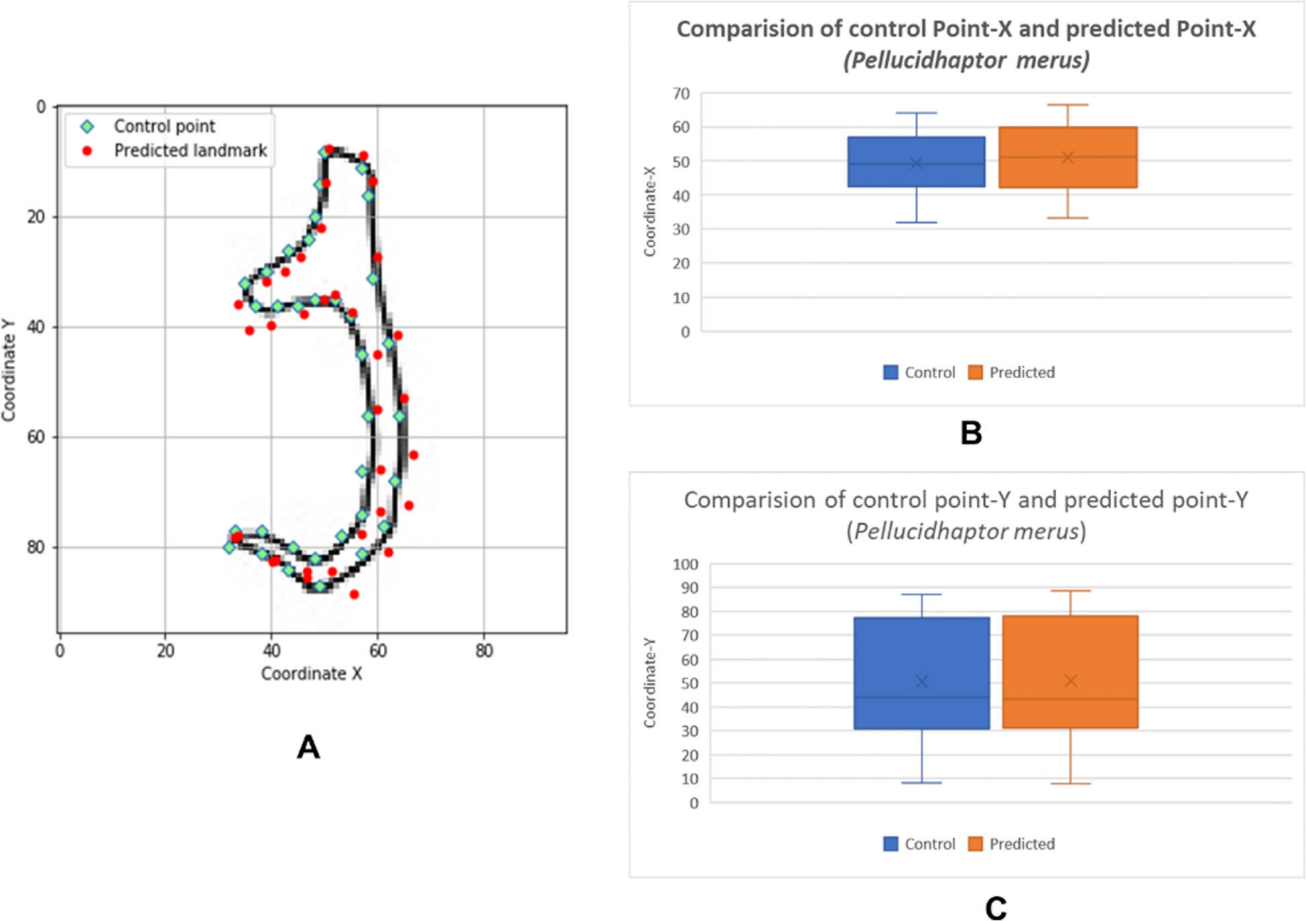
Comparison of control points and predicted points on 2D illustration of *Pellucidhaptor merus*. **a** Positions of control points (small green markers) and predicted points (red markers) on 2D illustration. **b** Boxplot for control point-X and predicted point-X (**c**) Boxplot for control point-Y and predicted point-Y

**Fig. 9:**
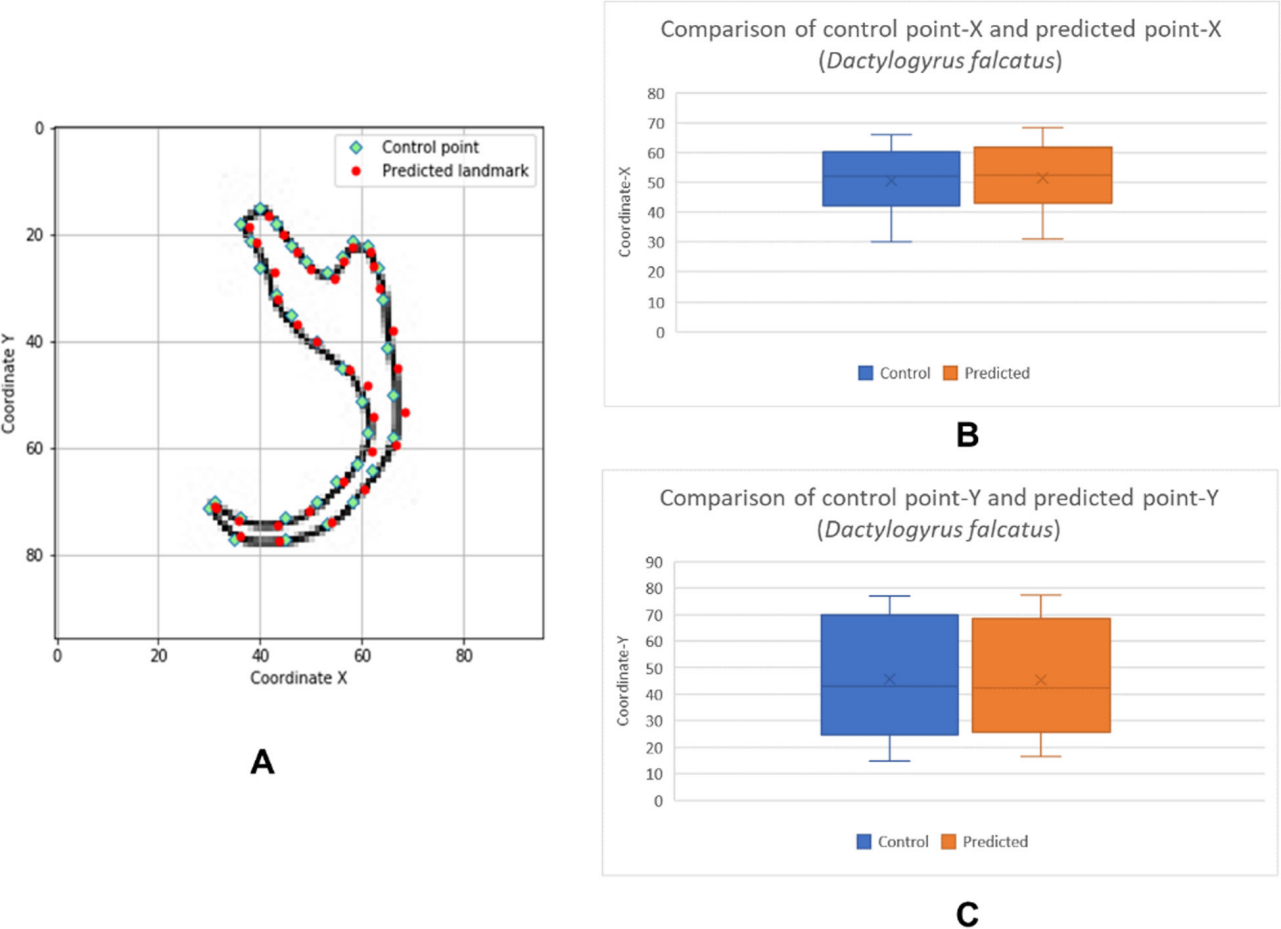
Comparison of control points and predicted points on 2D illustration of *Dactylogyrus falcatus*. **a** Positions of control points (small green markers) and predicted points (red markers) on 2D illustration. **b** Boxplot for control point-X and predicted point-X (**c**) Boxplot for control point-Y and predicted point-Y

**Fig. 10:**
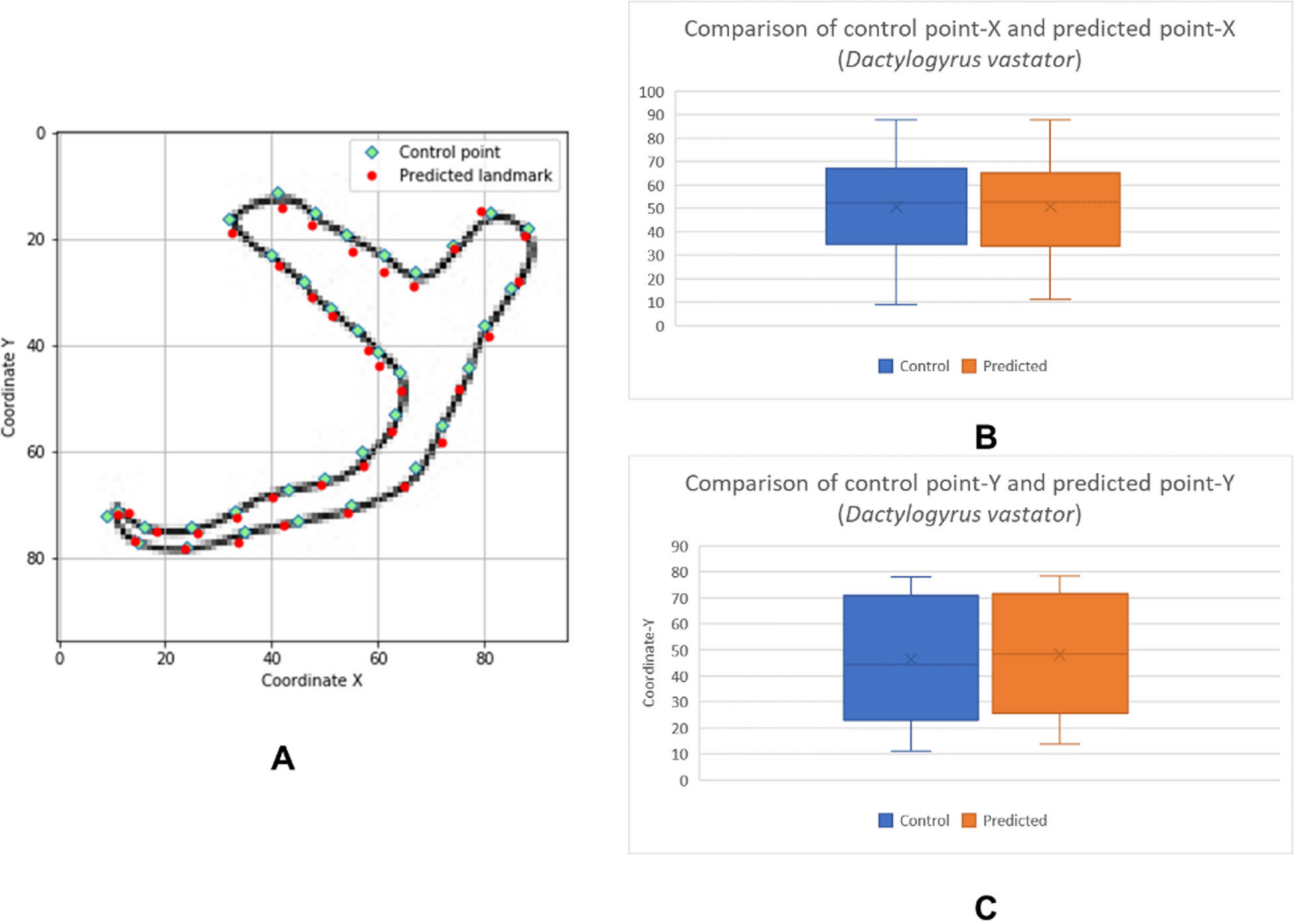
Comparison of control points and predicted points on 2D illustration of *Dactylogyrus vastator*. **a** Positions of control points (small green markers) and predicted points (red markers) on 2D illustration. **b** Boxplot for control point-X and predicted point-X (**c**) Boxplot for control point-Y and predicted point-Y

**Fig. 11:**
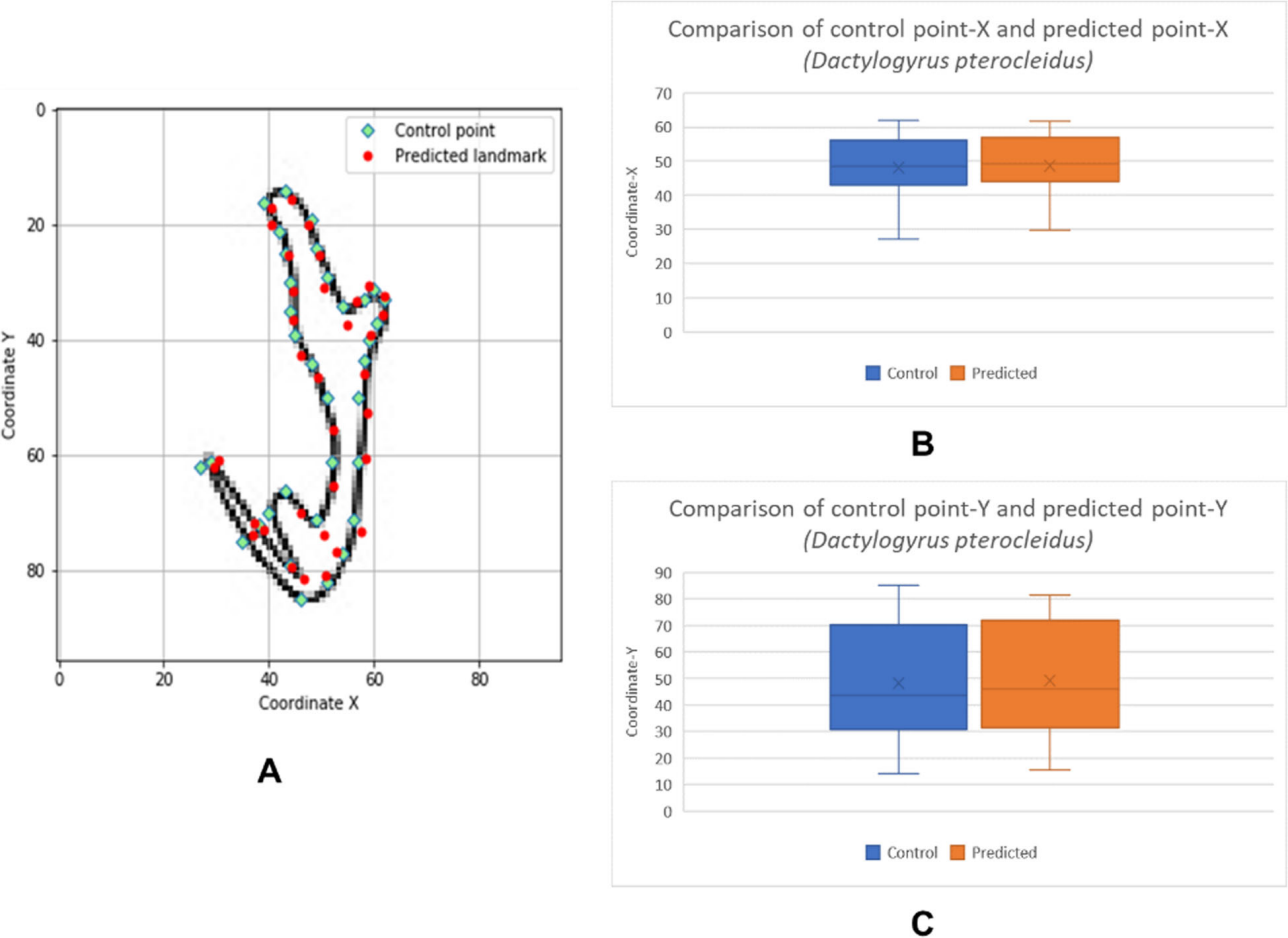
Comparison of control points and predicted points on 2D illustration of *Dactylogyrus pterocleidus*. **a** Positions of control points (small green markers) and predicted points (red markers) on 2D illustration. **b** Boxplot for control point-X and predicted point-X (**c**) Boxplot for control point-Y and predicted point-Y

**Fig. 12:**
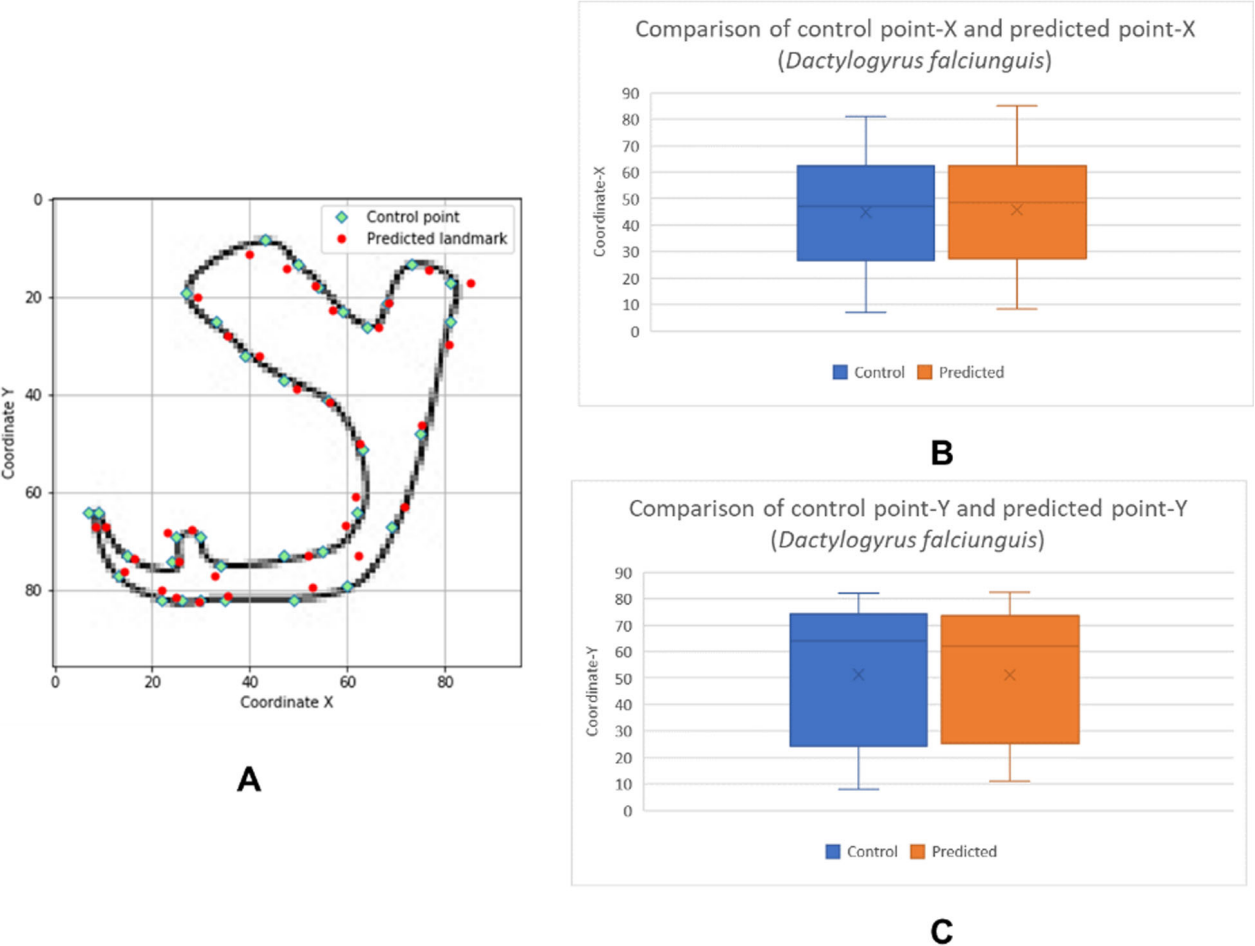
Comparison of control points and predicted points on 2D illustration of *Dactylogyrus falciunguis*. **a** Positions of control points (small green markers) and predicted points (red markers) on 2D illustration. **b** Boxplot for control point-X and predicted point-X (**c**) Boxplot for control point-Y and predicted point-Y

**Fig. 13:**
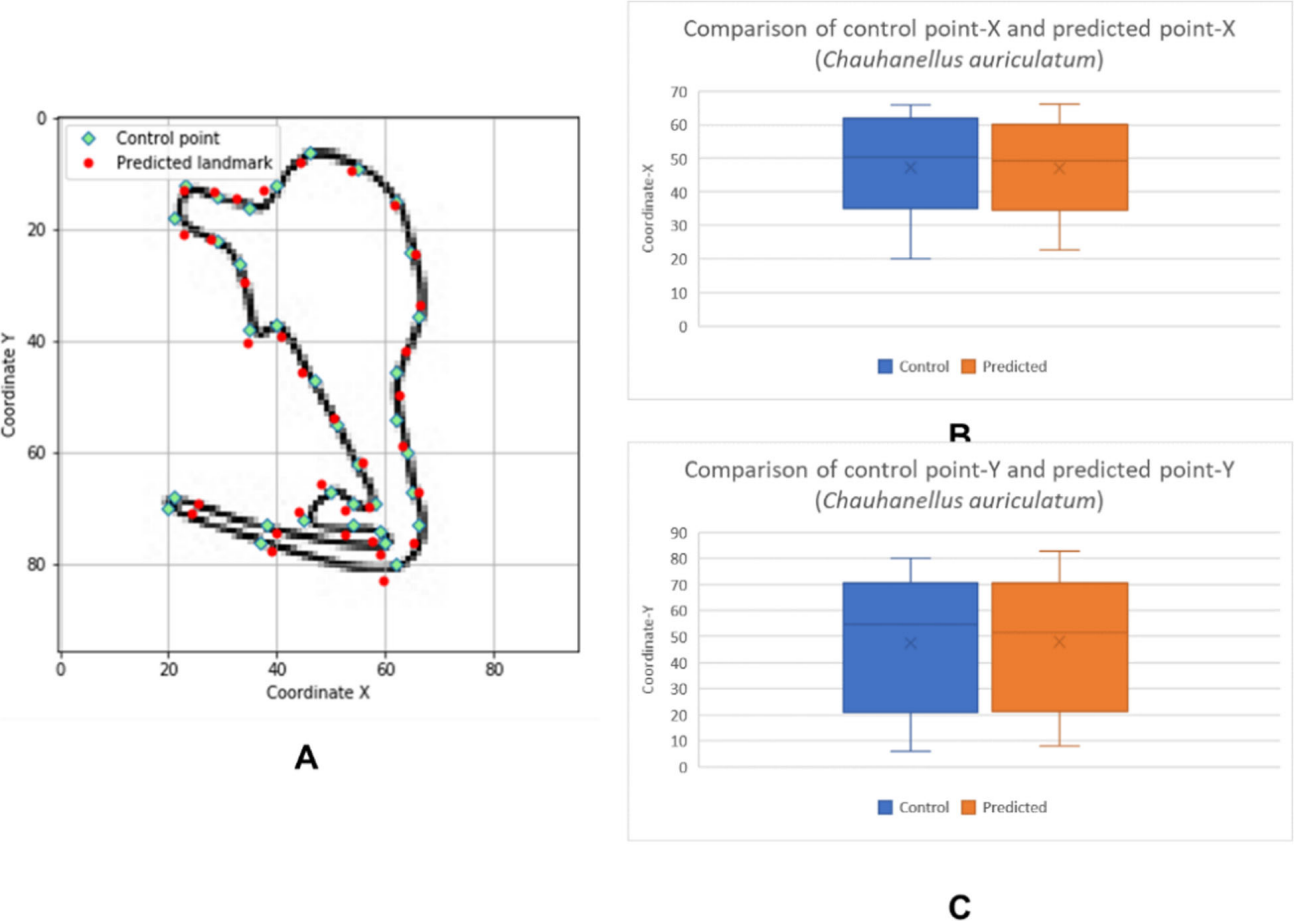
Comparison of control points and predicted points on 2D illustration of *Chauhanellus auriculatum*. **a** Positions of control points (small green markers) and predicted points (red markers) on 2D illustration. **b** Boxplot for control point-X and predicted point-X (**c**) Boxplot for control point-Y and predicted point-Y

**Fig. 14:**
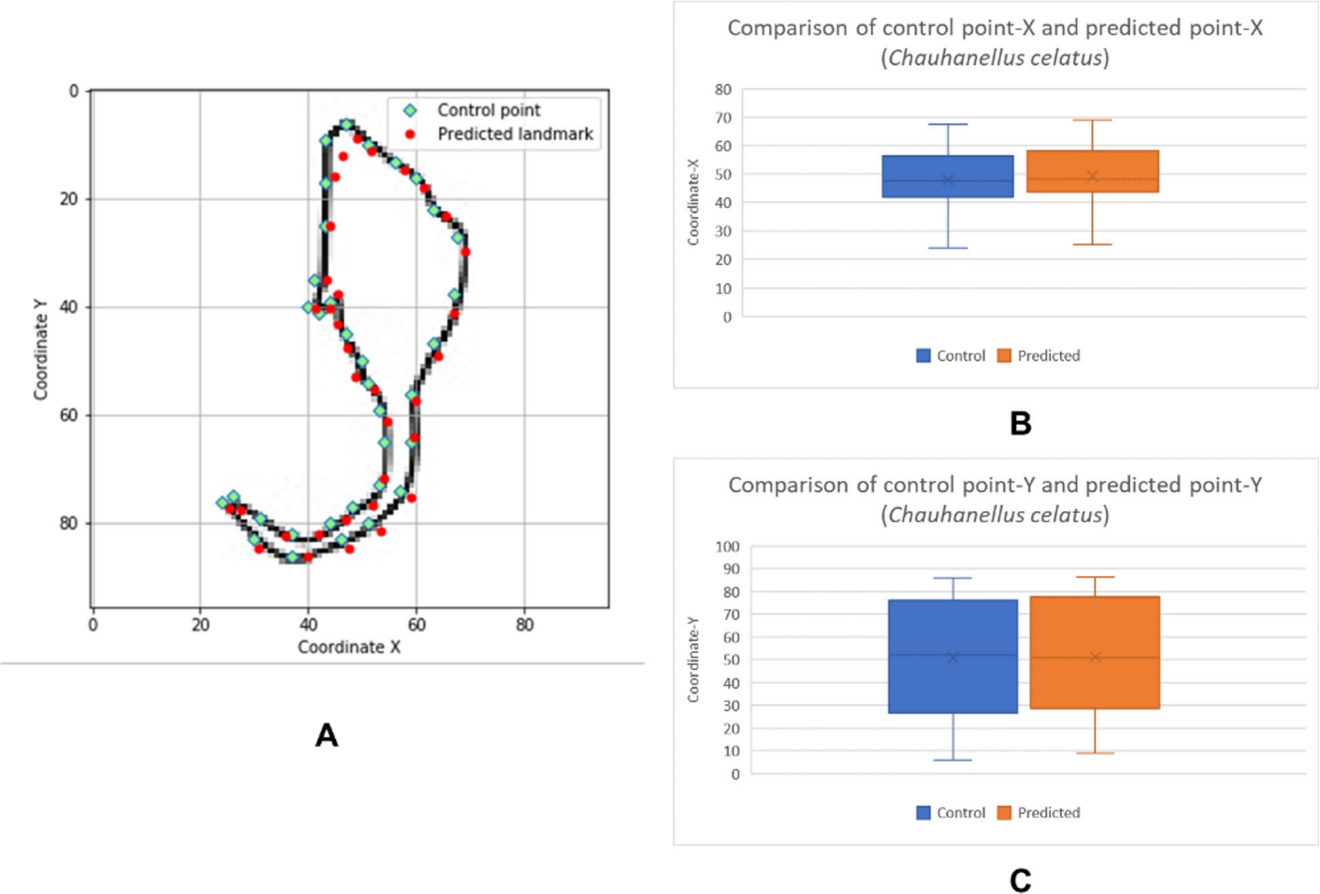
Comparison of control points and predicted points on 2D illustration of *Chauhanellus caelatus*. **a** Positions of control points (small green markers) and predicted points (red markers) on 2D illustration. **b** Boxplot for control point-X and predicted point-X (**c**) Boxplot for control point-Y and predicted point-Y

The box plots show the patterns of the control points and the predicted landmark points for each of the target monogenean anchors (Figs. 7, 8, 9, 10, 11, 12, 13 and 14). In each of the monogenean anchors, box plots for the distribution of coordinate-X (Figs. 7, 8, 9, 10, 11, 12, 13 and 14b) and coordinate-Y (Figs. 7, 8, 9, 10, 11, 12, 13 and 14c) were plotted separately. As shown in Figs. 7, 8, 9, 10, 11, 12, 13 and 14, all the box plots show a similar pattern of distribution between the control points and the predicted landmark points.

## Discussions

In this study, an automated 3D modeling pipeline empowered by a machine learning algorithm was developed. The box plots in Figs. 7, 8, 9, 10, 11, 12, 13 and 14 demonstrated the capability of the automated 3D modelling pipeline empowered by a machine learning algorithm in predicting the landmark points which can show a similar pattern of distribution with the control points. The distribution patterns presented in the box plots indicated that the predicted landmark points could be used for automating deformation of the generic 3D model to obtain the favorable 3D shape of anchors of the 8 selected monogenean species (Fig. 5). The box plots have verified the visual examination result as shown in Fig. 5 that the resulting 3D models can generally fit most parts of the 2D outline presented in the input illustrations of the anchor.

The automated 3D modelling pipeline developed in this study presents a user friendly way to automate the deformation of the generic 3D model into a target 3D anchor by using a 2D illustration as an input. This entire modelling pipeline offers a very straightforward way to construct a target 3D model with minimal human intervention. The automated 3D modelling approach presented in this study also improved the 3D modelling work published in Teo et al. [8], by obviating the usage of the comprehensive 3D modelling software, Autodesk 3ds Max to create a 3D model from scratch. To the best of our knowledge, this is the first time machine learning has been applied for landmark point detection for modeling a biological specimen. Application of machine learning for landmark point detection for modelling biological specimens is not reported in any published records.

However, the automated 3D modelling pipeline does not work very well on deriving 3D models of anchors with some intricate structures such as the extrusions found in the species of *D. pterocleidus* and *C. auriculatum* and the hook in *C. caelatus* (Fig. 6). Such distortion of shape is because these morphological structures (e.g. extrusions and hook) possess a relatively smaller surface area to be controlled by a higher number of point primitives compared with other regions of the anchor. This means a slight diversion of the predicted coordinates of those point primitives from the control points will cause an obvious distortion of shape.

The machine learning model was trained with the synthetic 2D illustrations of the anchor which are laterally positioned, and all the sizes and colors are standardized to 96 × 96 pixels and grayscale level. Hence, the model cannot effectively detect the landmark points on a wild image for auto-deformation of the generic 3D model to derive a target 3D shape. One must preprocess the input 2D illustration before it can be fetched into the automated 3D modelling pipeline to derive a target 3D model. Besides, it is also not feasible to use more than 5000 synthetic illustrations as training sets for machine learning in this study due to the limitation of the current infrastructure. As a result, the test accuracy level of the trained model is only limited up to approximately 80%.

As part of the future development, the entire project will be migrated to a cloud computing platform which can be harnessed to store the escalating number of training sets in a scalable cloud server while the powerful computing engine of cloud platform can be utilized to build and deploy the machine learning model for the automated 3D modelling pipeline [32]. In the long term, migration to the cloud platform is an essential move to scale up the development of the automated 3D modeling pipeline presented in this study. Finally, a generic 3D model will be created for each of the monogenean hard parts respectively and the training sets preparation and machine learning process will be repeated to train a more versatile machine learning model to detect landmark points on the input 2D illustrations of different monogenean hard parts.

## Conclusions

In this study, an automated 3D modelling pipeline was created using a machine learning technique, which is an Artificial Neural Network. The aim of this 3D modelling pipeline is to deform a digital generic 3D model of monogenean anchor into diverse forms of desired 3D anchors in an automated way. Despite some constraints and limitation as discussed above, the automated 3D modelling pipeline developed in this study has demonstrated a working idea of application of machine learning approach in a 3D modelling work. Besides, the development of this automated 3D modelling pipeline is also streamlined with some innovative methods to address some typical issues encountered in a machine learning related studies such as shortage of training set and big data storage management. In short, this study has not only developed an automated 3D modelling pipeline but also has demonstrated a cross-disciplinary research design that integrates machine learning into a specific domain of study such as 3D modelling of the biological structures.

## Methods

Prior to developing the entire automated 3D modelling pipeline empowered by a machine learning algorithm, a digital generic 3D model of an anchor was developed using a geometric 3D modelling method presented in a previous study [33]. This digital generic 3D model is used as a 3D template to derive another target 3D shape in the later stage. The development of the 3D modelling pipeline is presented in the following sub-sections.

### Data preparation for machine learning

In this study, the training data for machine learning are 2D illustrations of monogenean anchor. A well-trained machine learning model requires massive training data which can range from hundreds to millions to minimize the possibility of learning misleading or irrelevant patterns found in the training data [34]. In this study, 2D illustrations of monogenean anchor that could be extracted from existing publications were limited. It was also tedious to manually extract the 2D illustrations from publications to prepare the training set.

**Fig. 15:**
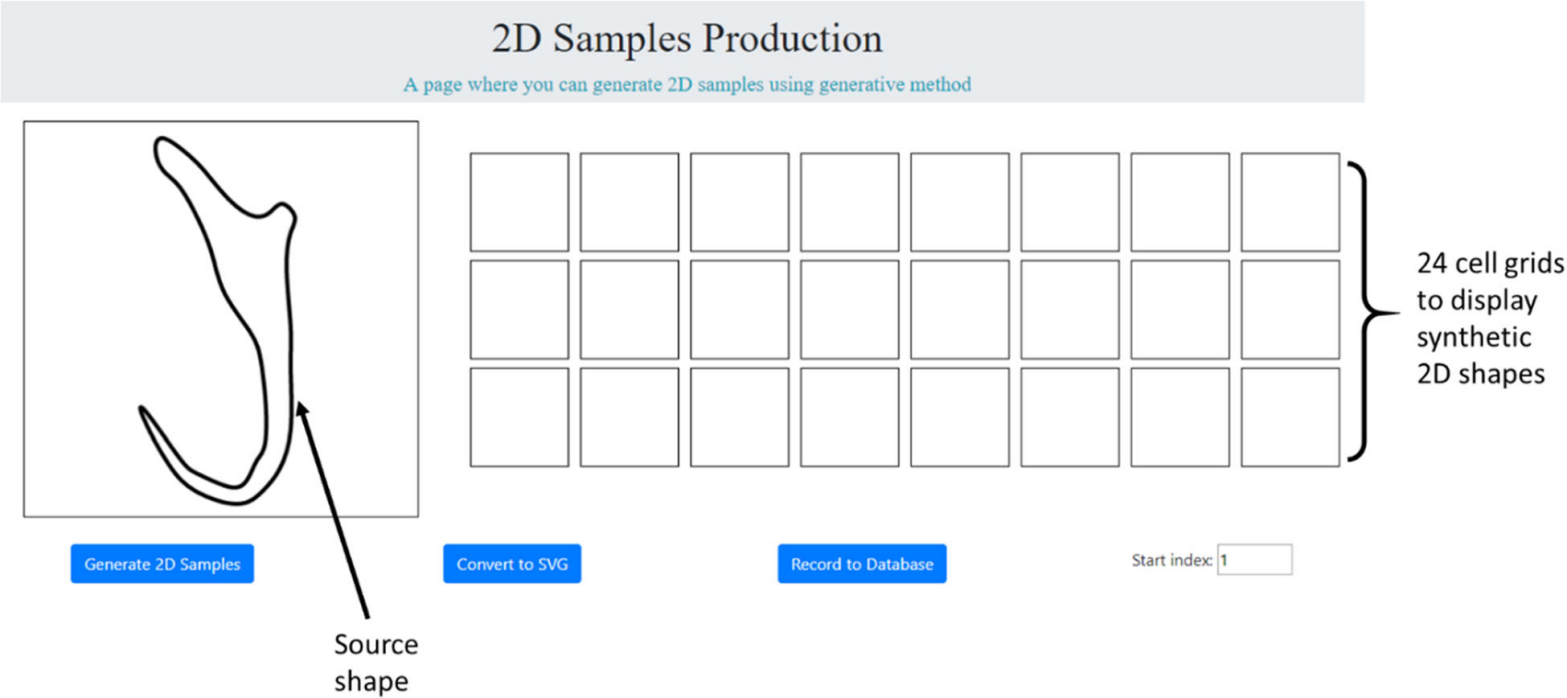
Data augmentation program to generate 2D illustrations of monogenean anchors

Hence, a semi-automated data augmentation approach was developed to generate a sequence of synthetic 2D illustrations of monogenean anchors. The core of this semi-automated data augmentation approach is a 2D shape interpolation algorithm to generate a sequence of interpolated shapes of the anchor as the synthetic illustrations from a process of morphing a source shape to a user-defined target shape. Pseudocode was drafted based on the 2D shape interpolation algorithm (See Additional file 1) and was used as a blueprint to develop a data augmentation program (Fig. 15). The data augmentation program was designed to generate 24 interpolated 2D shapes from one 2D source shape each time.

**Fig. 16:**
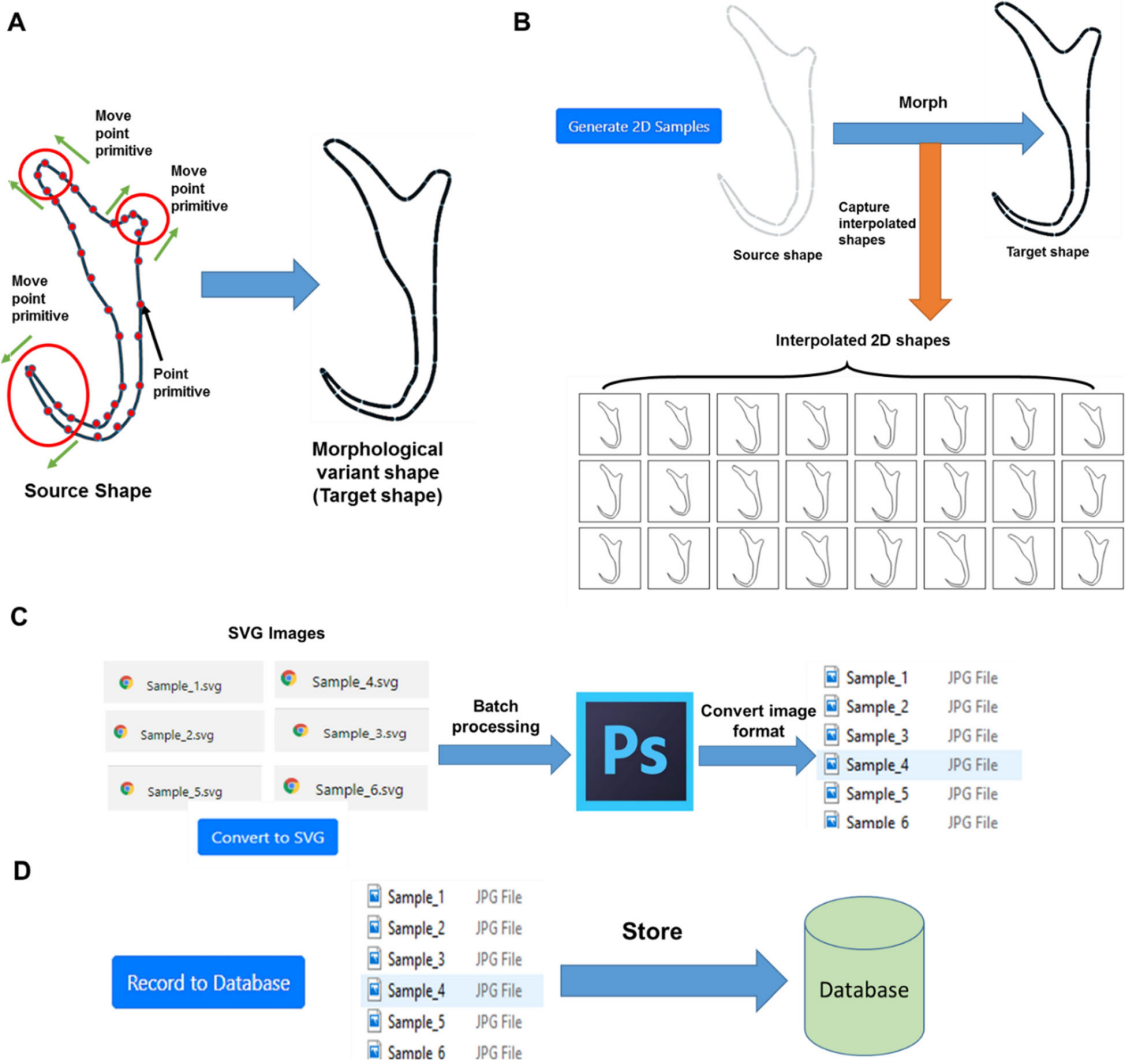
Workflow to generate synthetic 2D illustrations using data augmentation program. **a** Deform a source shape to a target shape, (**b**) Morph source shape to target shape, (**c**) Export 24 interpolated shapes to SVG images which will then undergo batch processing using Adobe Photoshop to convert them into JPEG format, (**d**) Store pixel values and associated 2D coordinates of each synthetic 2D illustration into database

**Fig. 17:**
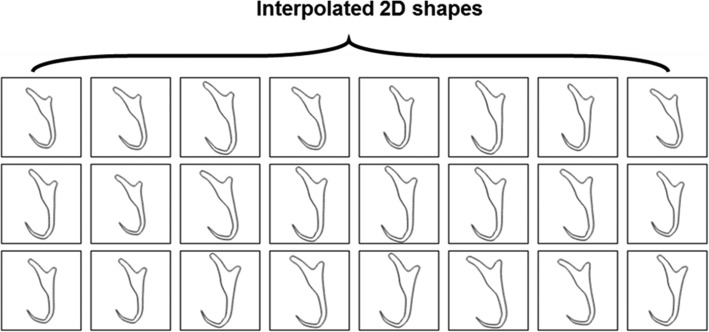
Twenty four interpolated 2D shapes

The procedure of using the data augmentation program to synthesize 2D illustration is as follows:

1. A 2D illustration of monogenean anchor extracted from a selected publication was used as the source shape. It was loaded into the data augmentation program
2. 2D point primitives on the source shape were selected individually and moved to a new position in the 2D Cartesian coordinate space to obtain a morphological variant shape which will be used as a target shape (Fig. 16a).
3. The button “Generate 2D Samples” was activated to morph the source shape to the target shape obtained from Step 2. While the process of morphing was on-going, a total of 24 interpolated 2D shapes were captured and displayed on the grid cells (Fig. 16b). Another enlarged cropped view of the 24 interpolated 2D shapes generated from the source shape is presented in Fig. 17.
4. Next, the button “Convert to SVG” was activated to export the captured interpolated 2D shapes to SVG images which will then be converted into JPEG format (Fig. 16c).
5. Finally, the button “Record to Database” button was activated to store the pixel values along with the associated 2D coordinates of the constituent point primitives of each synthetic 2D illustration into a database (Fig. 16d).

The data augmentation procedure described above was repeated to generate three sets of synthetic 2D illustrations with each of them consists of 1000, 2500 and 5000 datasets, respectively. Each set of synthetic 2D illustrations were used to train a machine learning model by following a supervised learning process as described below and the accuracy of the trained model is evaluated. This evaluation is to determine the number of datasets which is versatile enough to detect the landmark points on all types of target monogenean anchors in this study.

### Data storage

**Fig. 18:**
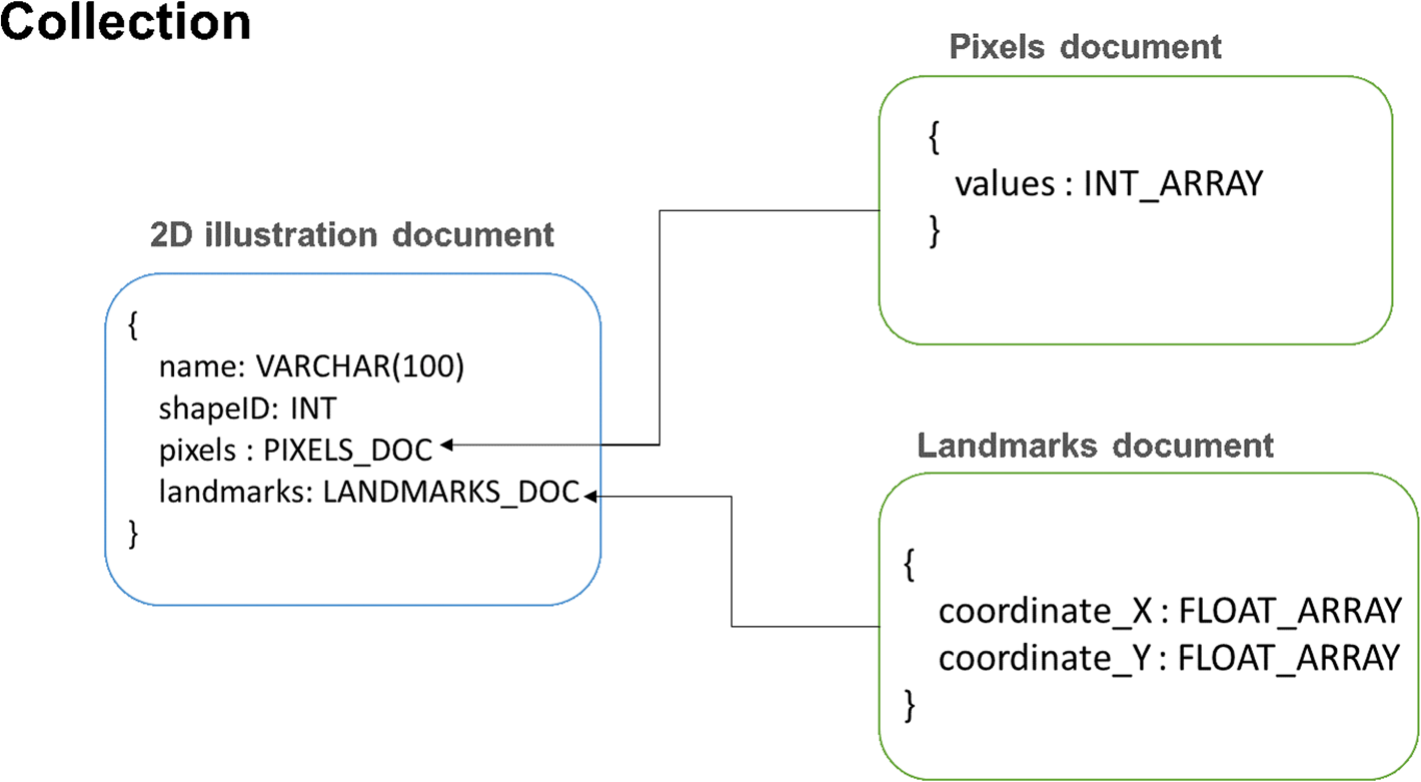
noSQL data model for illustration training set

A NoSQL database was developed using MongoDB (https://www.mongodb.com/) to store the training set in this study. The development of the database was initiated by defining a data model (Fig. 18) which is composed of a collection of documents which are “2D illustration document”, “Pixels document” and “Landmarks document”. In the MongoDB context, a collection is a container for structurally or conceptually similar documents whereas a document is a basic unit of data in MongoDB which encapsulates a group of related properties along with their associated values in a JSON object. In the data model, the “2D illustration document” was defined as a parent node to hold all the relevant 2D illustration information in a JSON object, which consists of four properties which are “name”, “shapeID”, “pixels” and “landmarks”. The “name” property was defined to hold an illustration identifier with a maximum of 100 characters whereas the “shapeID” is a numerical type property to hold the type of 2D shape. The “pixels” and “landmarks” properties were defined to hold the “Pixels document” and “Landmarks document” respectively. The “Pixels document” and “Landmark document” can be considered as two child nodes associated with the parent node, “2D illustration document”. In the child node “Pixels document”, there is only one property defined to hold an integer array of pixel values whereas there are two properties defined in “Landmark document” to hold an array of 2D coordinates, “coordinate_X” and “coordinate_Y” of each constituent point primitives that form a synthetic illustration.

### Machine learning model

An Artificial Neural Network (ANN) was trained to enable landmark detection on an input illustration. The ANN was based on a multilayer perceptron architecture adapted from a landmark detection study [35]. The input layer consists of 9216 nodes which hold an array of 96 × 96 pixels values of every 2D illustration training set. The number of hidden layers and the number of nodes in each of the hidden layers were set by a trial and error process started with a single hidden layer and a multiple of 128 nodes (128, 256, and 512) in each hidden layer. In the end, the network architecture comprising of two hidden layers with 512 nodes and 128 nodes, respectively, was adopted as it generally showed the decent performance of landmark detection. In the hidden layers, the ReLU was used as the activation function to calculate the weighted sum of the input nodes because it is one of the popular choices for a regression problem that aims to predict any positive continuous value [34, 36]. In the output layer are the 68 positive continuous values which denote predicted 2D coordinates (x, y) for 34 point primitives. These predicted point primitives are regarded as the landmark points in the context of this study. The trained neural network is aimed to detect these landmark points on an input 2D illustration.

**Fig. 19:**
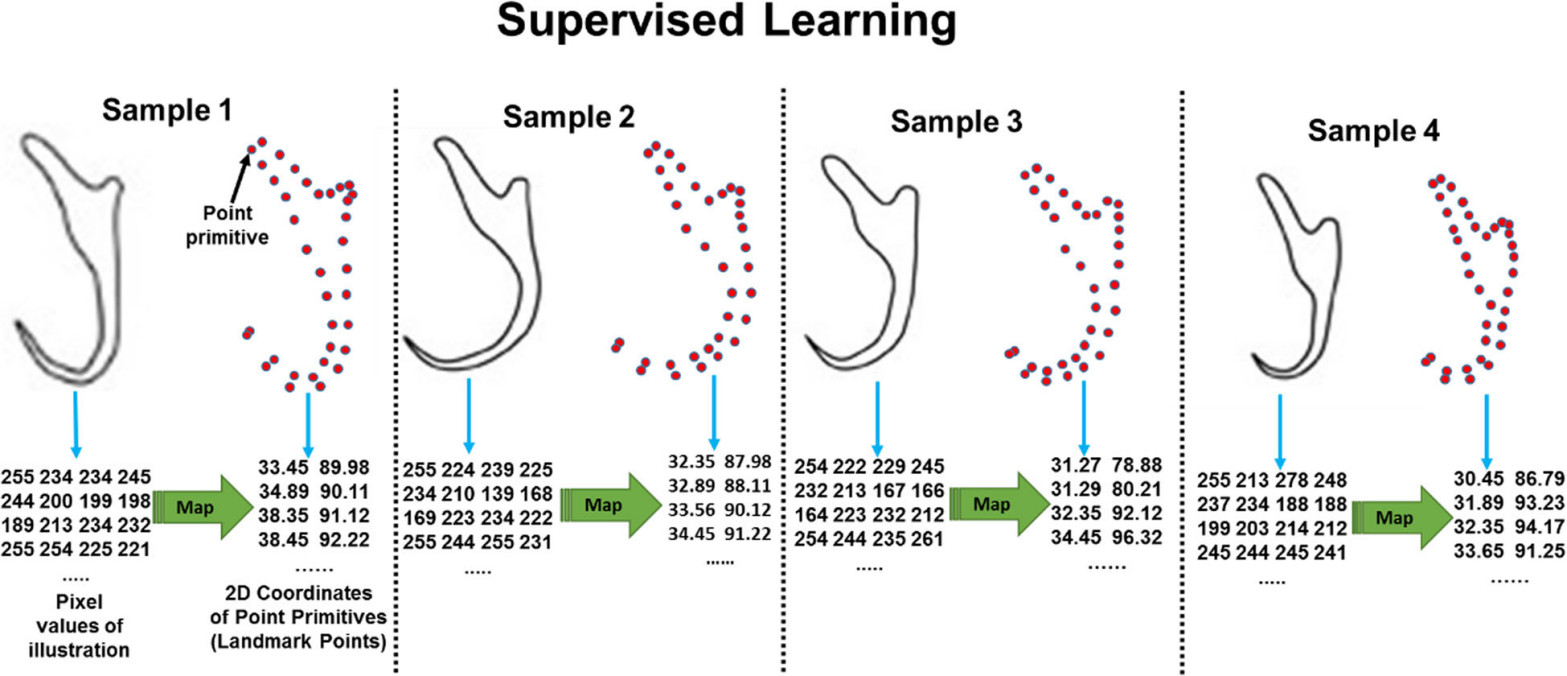
Supervised learning to map pixel values of illustrations (from four selected samples) to each of their associated 2D coordinates of constituent point primitives

The ANN was trained via a supervised learning process by mapping the pixel values of all the synthetic illustrations retrieved from the MongoDB database to each of their associated 2D coordinates of constituent point primitives (Fig. 19). Before training the ANN, all pixel values samples were divided into a training set (80% of samples) and a test set (20% of samples). The test set contains independent samples which are used to evaluate the accuracy of the trained model in a later stage. Besides, 30% of the samples were set apart from the training set to be used for model validation during the training process.

The ANN was trained for 200 epochs in mini-batches of 20 samples by using Mean Square Error (MSE) as the loss function and Stochastic Gradient Descent (SGD) as the optimizer to repeatedly update the weight parameters of the preliminary machine learning models. The hyperparameters adapted from [35] as follows was used to configure the optimizer:

- Learning rate = 0.01
- Momentum = 0.9
- Decay rate = 0
- Nesterov momentum = True

### Formation of automated 3D modelling pipeline

**Fig. 20:**
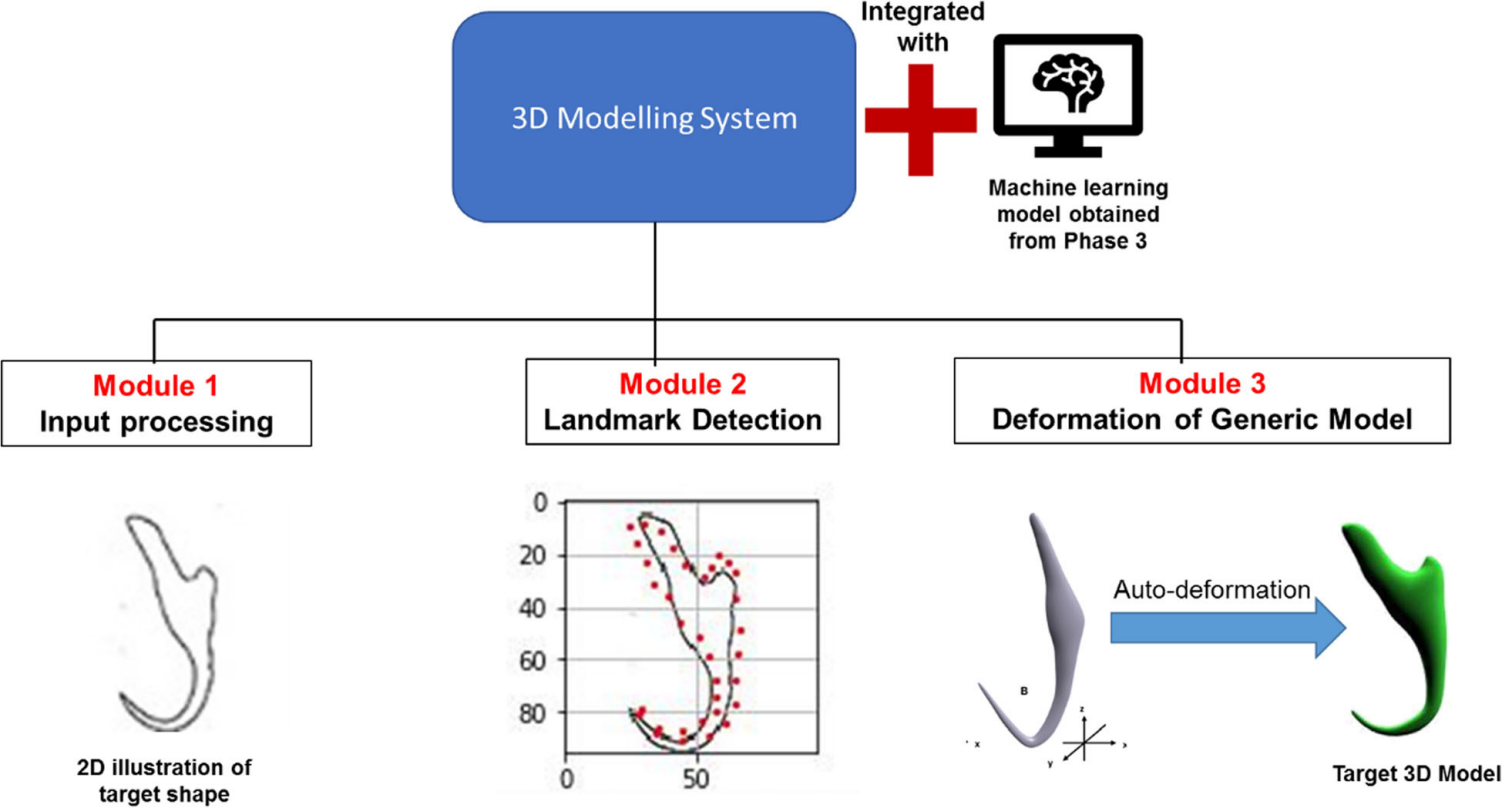
3D modelling system design

**Fig. 21:**
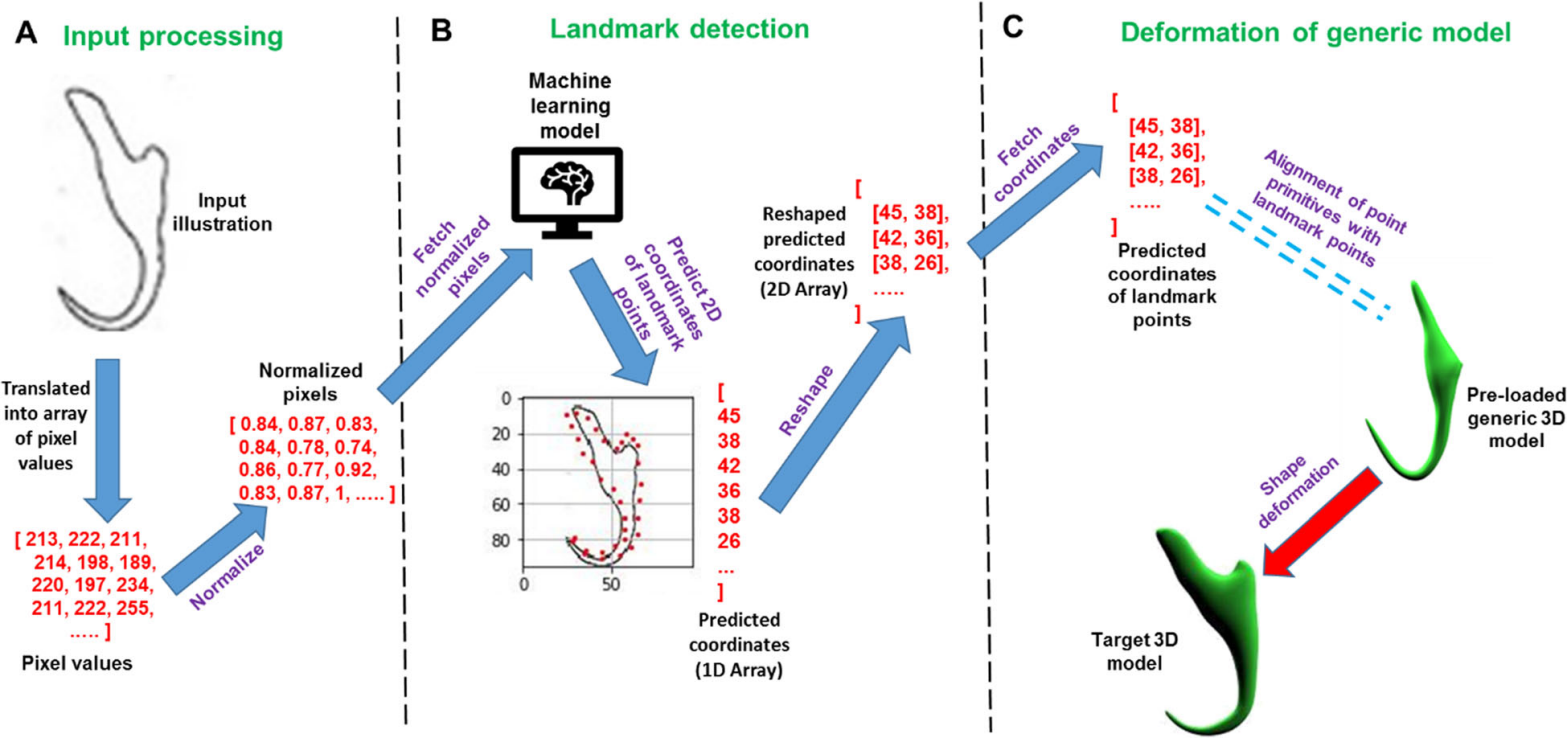
Automated 3D modelling pipeline. **a** Input processing, (**b**) Landmark localization, (**c**) Deformation of generic 3D model

In this step, a 3D modelling system which is composed of three modules was developed (Fig. 20). The first module is to read and process the input illustration by translating it into an array of normalized pixel values (Fig. 21a). The second module is to detect the 34 landmark points on the input illustration through the trained model (Fig. 21b). The third module is to deform the digital generic 3D model by aligning the constituent point primitives of the generic model with a corresponding landmark point signified by the predicted coordinate values obtained from the second module (Fig. 21c). These three modules along with the trained machine learning model work synergistically to form an automated 3D modelling pipeline which can automatically deform the digital generic 3D model into a target 3D shape by using a 2D illustration as an input.

**Fig. 22:**
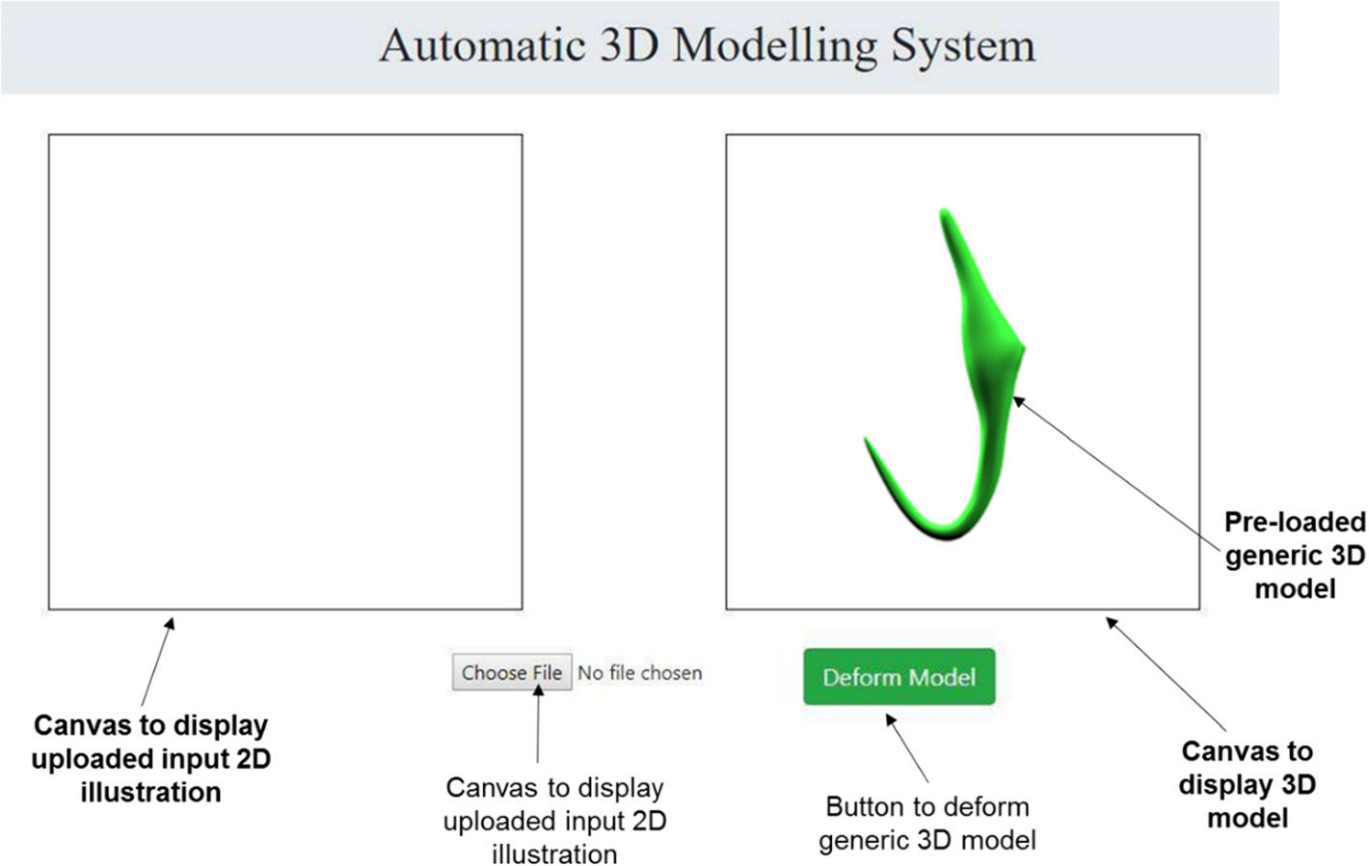
The automatic 3D modelling system

**Fig. 23:**
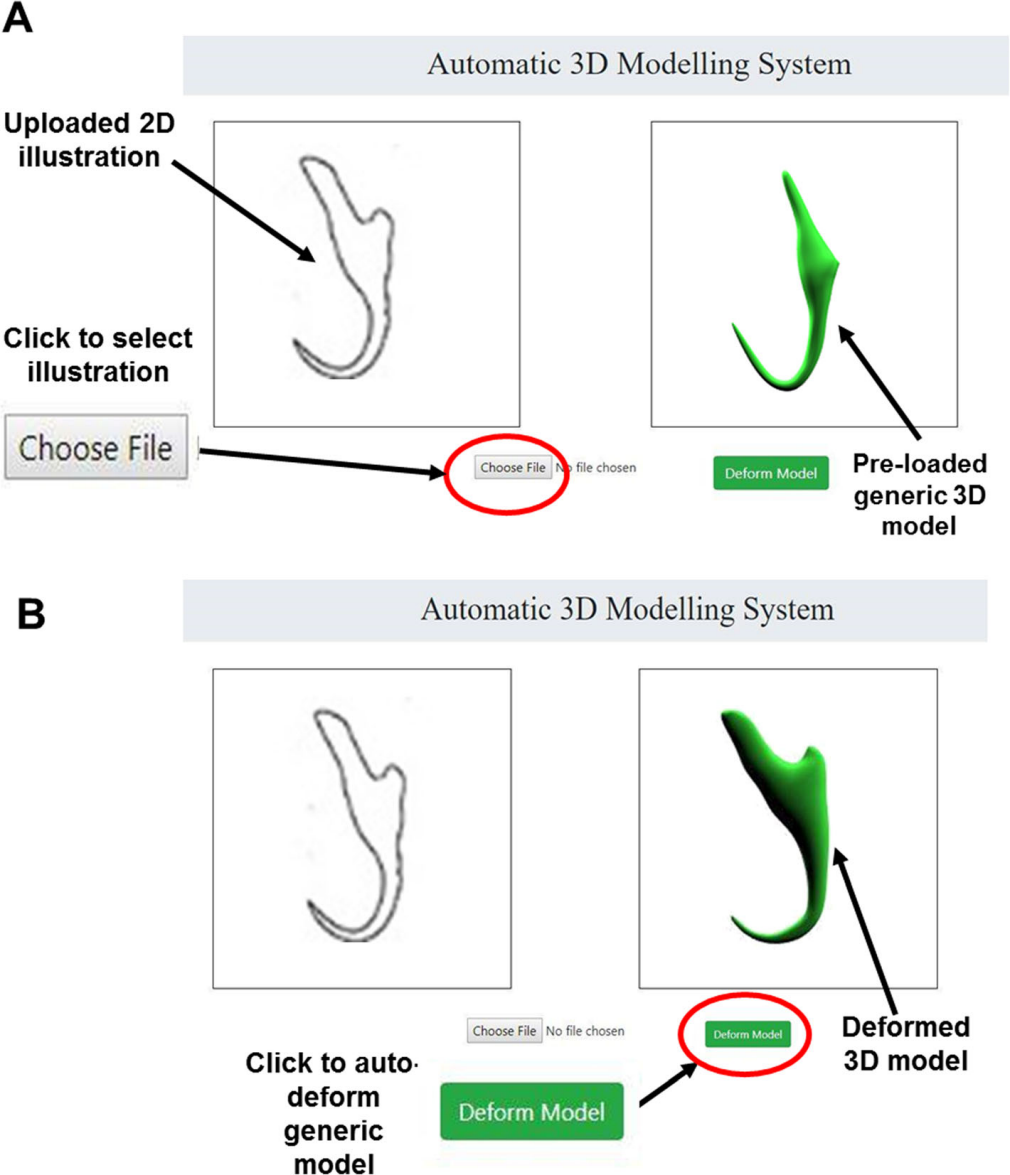
Implementation of the automated 3D modelling pipeline. **a** Upload 2D illustration, (**b**) Generate 3D model through the automated 3D modelling pipeline

A pseudocode which features the operating logic of the automated 3D modelling pipeline was drafted (See Additional file 2) and this pseudocode was used as a blueprint to develop the 3D modelling system which is a full stack web application (Fig. 22). The procedures of using the developed 3D modelling system to implement the automated 3D modelling pipeline are as follows:

1. A 2D illustration of a selected monogenean anchor was uploaded onto the system interface. This was done by activating the “Choose File” button and followed with selecting a 2D illustration as the target shape. (Fig. 23a)
2. Next, the button “Deform Model” was activated (Fig. 23b) to fetch the input 2D illustration to the back-end components of this system to enable it to go through the entire 3D modelling pipeline (input processing, landmark detection and deformation of the generic model).

The procedure of using the 3D modelling system as described above was repeated to generate the eight selected monogenean anchors through the automated 3D modelling pipeline and the results are presented in the following section.

### 3D model evaluation

**Fig. 24:**
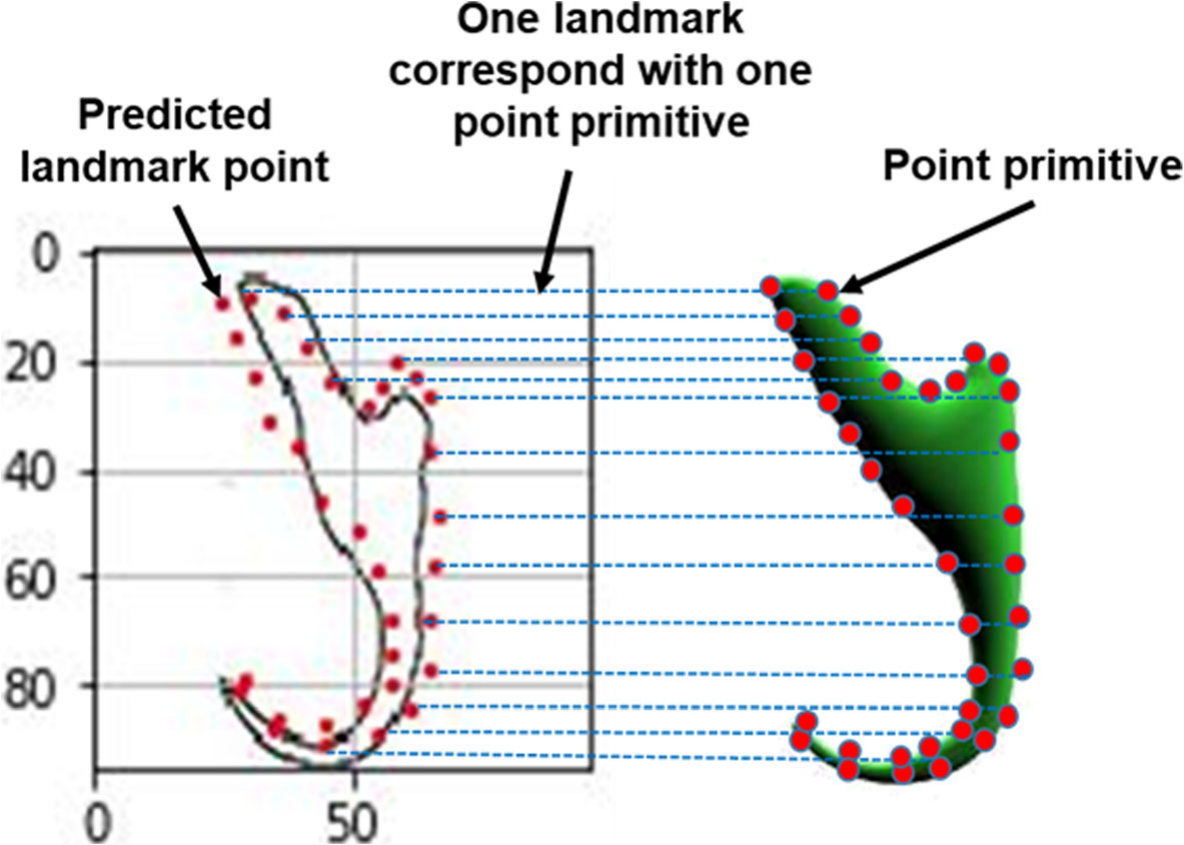
Dependency of the target 3D shape on predicted landmark point position

The evaluation of the resulting 3D model was based on a quantitative analysis which was done by plotting box plots to compare the distribution of the control points and the predicted landmark points for each of the target monogenean anchors. The shape of the target 3D model produced by the automated 3D modelling pipeline is dependent on the landmark points predicted by the machine learning model which empowers the 3D modelling system (Fig. 24). Ideally, all the predicted landmark points should be positioned along the edge of the 2D illustration so that this can result in an optimum 3D shape which can match with the target shape presented in the input 2D illustration.

**Fig. 25:**
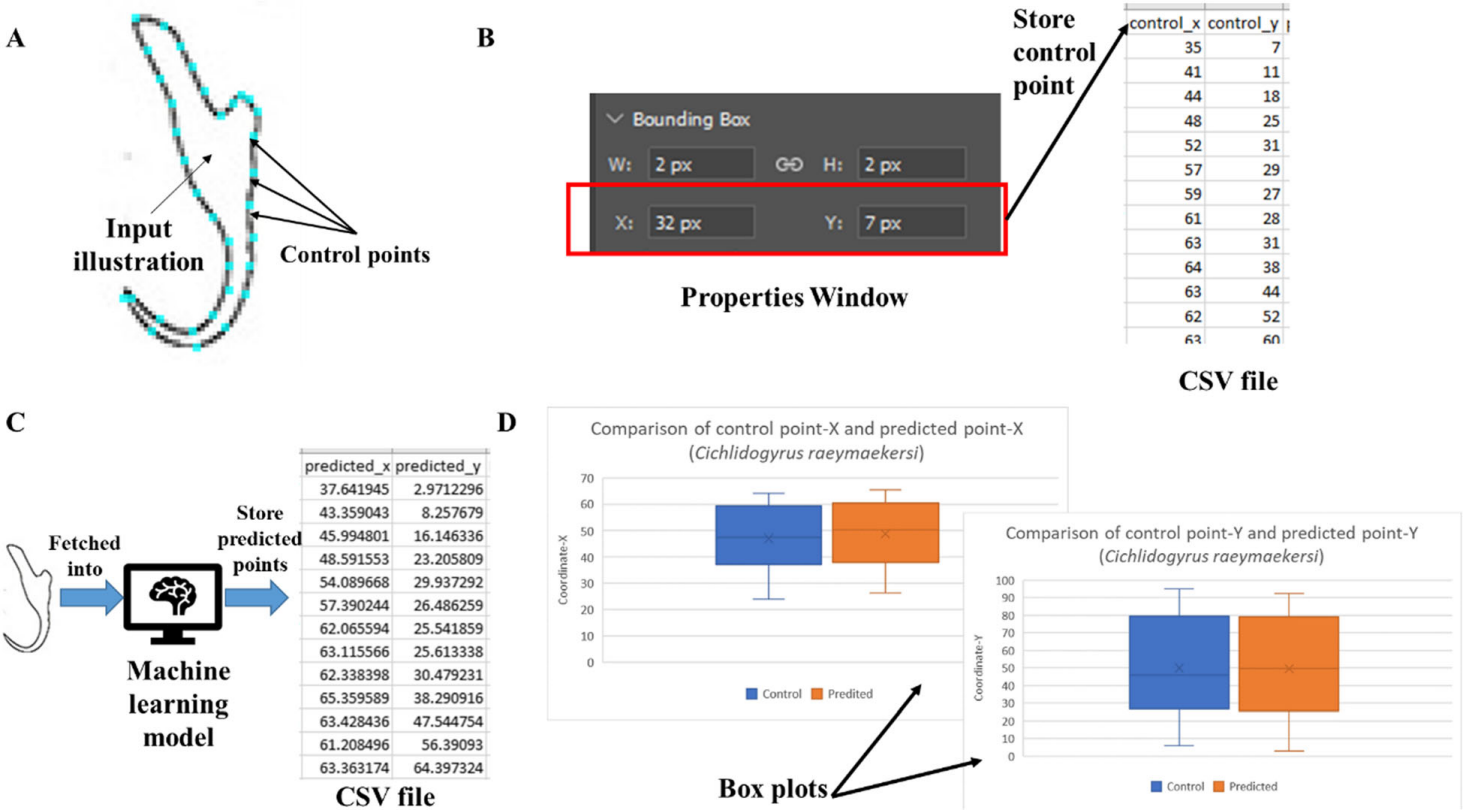
Procedures of creating box plots to examine disparity pattern of control points and landmark points. **a** Manual annotation of control points on an input 2D illustration. **b** Extract coordinates of control points from the Properties Window in Adobe Photoshop. **c** Collect predicted landmark point from the machine learning model. **d** Create two box plots using Microsoft Excel

Based on this understanding, the steps below are followed:

1. A sequence of control points were manually annotated on the ideal positions along the edge of an input 2D illustrations (Fig. 25a).
2. The coordinates of the control points were extracted from the Properties Window in Adobe Photoshop and were stored in a CSV file (Fig. 25b).
3. The same input 2D illustration was fetched to the 3D modelling system and the landmark point coordinates predicted by the machine learning model were collected and stored in a CSV file (Fig. 25c).
4. The Coordinate-X and Coordinate-Y of the control points and the predicted landmark points were loaded to create two box plots (Fig. 25d). The disparity pattern of the Coordinate-X and Coordinate-Y between the control points and the predicted landmark points were examined and analyzed.
5. Steps I-IV were repeated for all the 8 input 2D illustrations used for creating the target 3D anchors.

## Supplementary information

**Additional file 1.** Pseudocode for 2D shape interpolation algorithm.

**Additional file 2.** Pseudocode for the development of automatic 3D modelling system.

## Data Availability

The datasets used and/or analysed during the current study are available from the corresponding author on reasonable request.

## Abbreviations

ANN: Artificial Neural Network
CNN: Convolutional Neural Network
RNN: Recurrent Neural Network
SEM: Scanning Electron Microscope
