# Supplementary material for: An automated 3D modeling pipeline for constructing 3D models of MONOGENEAN HARDPART using machine learning techniques
Source: BMC Bioinformatics. 2019 Dec 24;20(Suppl 19):658. doi: 10.1186/s12859-019-3210-x (PMC6929343; doi:10.1186/s12859-019-3210-x)
Supplement: Supplementary file 1 — Additional file 1. Pseudocode for 2D shape interpolation algorithm. [file 12859_2019_3210_MOESM1_ESM.pdf]

Input

- 2D coordinates of point primitives to form a 2D source shape

Initialization

- Declare array data structure to hold 2D coordinates input

Build 2D vector graphic of the source shape using 2D coordinates from array

- For each 2D coordinates in array
  - Define one 2D point
  - Add 2D point to a polygonal chain of source shape, *SourceShape* $[(x_1, y_1), (x_2, y_2), (x_3, y_3), \ldots, (x_i, y_i)]$

- Smoothen *SourceShape* using in-built *smooth* function

Build 2D vector graphic of a target shape by moving 2D points of source shape

- On mouse drag, get mouse cursor position, *mouseX, mouseY*
- On mouse up, set mouse cursor position as 2D point new position, *newX, newY*
  - *(newX, newY) = (mouseX, mouseY)*
- Track index of 2D point being moved.
- Calculate delta between newPoint position and source point at position index, *i*
  - Delta X, $\Delta x = | newX - x_i | * 4$
  - Delta Y, $\Delta y = | newY - y_i | * 4$
- Store Δx and Δy in *deltaX_Arr* and *deltaY_Arr* array respectively

Interpolate 2D shape

- For each Δx and Δy in *deltaX_Arr* and *deltaY_Arr*
  - set $dx = \Delta x_j / (24 * 4)$;
  - set $dy = \Delta y_j / (24 * 4)$;
  - set interpolated coordinate X, $inter\_x = x_j + dx * j$
  - set interpolated coordinate Y, $intex\_y = y_j + dy * j$
  - Define a new 2D point using *inter_X* and *inter_Y* coordinates
  - Add the new 2D point to a polygonal chain of interpolated shape, *InterpolateShape* $[(inter\_x_1, inter\_y_1), (inter\_x_2, inter\_y_2), (inter\_x_3, inter\_y_3), \ldots, (inter\_x_i, inter\_y_i)]$
- Smoothen *InterpolateShape* using in-built *smooth* function

Random transformation on interpolated 2D shape

- Declare scale factor array, *scaleFactor* with initialized factors, [0.85, 0.88, 0.9, 1, 1.02, 1.04, 1.05]
- Declare rotation degree array, *rotateDegree* with initialized degrees [-10, -7, -5, 0, 2, 5, 8, 10]
- For each *InterpolateShape*
  - Get random index, *randIndex*, from random generator
  - Apply *scaleFactor* at *randIndex*
  - Apply *rotationDegree* at *randIndex*
