# Supplementary material for: An automated 3D modeling pipeline for constructing 3D models of MONOGENEAN HARDPART using machine learning techniques
Source: BMC Bioinformatics. 2019 Dec 24;20(Suppl 19):658. doi: 10.1186/s12859-019-3210-x (PMC6929343; doi:10.1186/s12859-019-3210-x)
Supplement: Supplementary file 2 — Additional file 2. Pseudocode for the development of automatic 3D modelling system. [file 12859_2019_3210_MOESM2_ESM.pdf]

Module 1: Receiving and processing Input

- Declare variable, *img*, to hold pixel values of input illustration
- Read image file as an array of pixel values and assign it to *img* variable
- Normalize pixel values of input illustration, using formula:
    - set *norm_img = img / 255*
- Transform normalize pixel values into 2D array
    - *norm_img* *2D Tensor with shape(1, 96 * 96)*

Module 2: Landmark Localization

- Load machine learning model (a h5 file) into system
- Fetch 2D array of *norm_img* into machine learning model
    - *model.predict(2D tensor norm_img)*
- Declare a variable, *predicted_output,* to hold the values of predicted landmark coordinates
    - Set *predicted_output = model.predict(2D tensor norm_img)*
- Reshape 68 numerical output values into 34 pairs of coordinates and add them into a two-dimensional array, *coordinate_array*
    - For each 68 *predicted_output* values with index, i = 0, 1, 2, 3…67

```
if index is even
    Set coords_x = predicted_output
if index is odd
    Set coords_y = predicited_output

coordinate_array.push(coords_x, coords_y)
```

Module 3: Deformation of generic model

- Set a 3D scene
- Load the generic 3D model from Phase 1 into 3D scene
- Align coordinates of point primitives of generic 3D model, point_primitive_x & point_primitive_y, with *coordinate_array* obtained in previous step.
  (This step shall deform the generic 3D model)
    - For each coords_x , coords_y in coordinate_array with index, i = 0, 1, 2, 3…67
        - Set $point\_primitive\_x_i = coords\_x_i$
        - Set $point\_primitive\_y_i = coords\_y_i$
- Smoothen deformed 3D model using Catmull_Clark modifier to obtain final 3D mesh
- Add smoothened 3D model to 3D scene
